# A pragmatic randomized waitlist-controlled effectiveness and cost-effectiveness trial of digital interventions for depression and anxiety

**DOI:** 10.1038/s41746-020-0293-8

**Published:** 2020-06-15

**Authors:** Derek Richards, Angel Enrique, Nora Eilert, Matthew Franklin, Jorge Palacios, Daniel Duffy, Caroline Earley, Judith Chapman, Grace Jell, Sarah Sollesse, Ladislav Timulak

**Affiliations:** 1 University of Dublin, Trinity College, School of Psychology, E-mental Health Research Group, Dublin, Ireland; 2 Clinical Research & Innovation, SilverCloud Health, Dublin, Ireland; 3 HEDS, ScHARR, University of Sheffield, Sheffield, England; 4 Berkshire Healthcare NHS Foundation Trust, London, Berkshire, England

**Keywords:** Health services, Health care economics

## Abstract

Utilization of internet-delivered cognitive behavioural therapy (iCBT) for treating depression and anxiety disorders in stepped-care models, such as the UK’s Improving Access to Psychological Therapies (IAPT), is a potential solution for addressing the treatment gap in mental health. We investigated the effectiveness and cost-effectiveness of iCBT when fully integrated within IAPT stepped-care settings. We conducted an 8-week pragmatic randomized controlled trial with a 2:1 (iCBT intervention: waiting-list) allocation, for participants referred to an IAPT Step 2 service with depression and anxiety symptoms (Trial registration: ISRCTN91967124). The primary outcomes measures were PHQ-9 (depressive symptoms) and GAD-7 (anxiety symptoms) and WSAS (functional impairment) as a secondary outcome. The cost-effectiveness analysis was based on EQ-5D-5L (preference-based health status) to elicit the quality-adjust life year (QALY) and a modified-Client Service Receipt Inventory (care resource-use). Diagnostic interviews were administered at baseline and 3 months. Three-hundred and sixty-one participants were randomized (iCBT, 241; waiting-list, 120). Intention-to-treat analyses showed significant interaction effects for the PHQ-9 (*b* = −2.75, 95% CI −4.00, −1.50) and GAD-7 (*b* = −2.79, 95% CI −4.00, −1.58) in favour of iCBT at 8-week and further improvements observed up to 12-months. Over 8-weeks the probability of cost-effectiveness was 46.6% if decision makers are willing to pay £30,000 per QALY, increasing to 91.2% when the control-arm’s outcomes and costs were extrapolated over 12-months. Results indicate that iCBT for depression and anxiety is effective and potentially cost-effective in the long-term within IAPT. Upscaling the use of iCBT as part of stepped care could help to enhance IAPT outcomes. The pragmatic trial design supports the ecological validity of the findings.

## Introduction

Stepped-care models have been proposed as a potential solution^1^ to bridge the substantial gap between the prevalence of common mental health disorders, including depression and anxiety, and the access rates for evidence-based treatments^2,3^. Stepped-care seeks to up-scale treatment initiatives by matching treatment intensity and duration to clients’ presenting needs, thereby optimizing outcomes and service capacity utilization. Investing in upscaling initiatives for mental health treatments is projected to produce large returns at a benefit-to-cost ratio of 3.3–5.7 to 1 when accounting for economic benefits and the value of health returns^4^. The Improving Access to Psychological Therapies (IAPT) programme in the UK is one of the first examples of a mental health stepped-care model implemented nationwide^5^.

IAPT services offer evidence-based treatments to individuals experiencing depression and/or anxiety, providing low-intensity interventions alongside traditional treatments (e.g. face-to-face therapy)^5^. Specifically, at Step 2, low-intensity interventions are offered to patients presenting with mild to moderate depression and anxiety symptoms, while those with more severe or complex presentations of depression and anxiety are assigned to step 3 high-intensity treatments. Low-intensity interventions include empirically established treatments like guided bibliotherapy and internet-delivered cognitive behavioural therapy (iCBT). Similar multicomponent models of care exist in other countries, such as the collaborative care models in the USA^6^. A key aspect of IAPT is routine outcome monitoring, which is used to improve individual clinical outcomes by aiding ongoing treatment decisions, but also to establish publicly available service-level clinical performance reports^7^. In the period 2018–19, IAPT received 1.6 million new referrals, of which 1.09 million were seen at least once for assessment and guidance and 582,556 received a course of therapy (defined as two or more sessions)^8^.

Political and policy initiatives that helped establish IAPT promised significant economic benefits, claiming that making evidence-based psychological treatments available would have no net cost to the Treasury^9^, yet envisioned economical returns from IAPT remain debated^10^. Internet-delivered interventions may be one way to improve IAPT outcomes in a cost-effective way^11^. However, currently iCBT accounts for only 7% of treatments completed within IAPT^8^. Clark et al. found that amongst services that achieve lower treatment rates, engaging more users in treatment could improve recovery and reliable improvement by 33% and 90%, respectively^7^, highlighting the potential for increased use of iCBT within IAPT to help achieve this aim.

Generally, iCBT for depression and anxiety has been found to significantly reduce symptoms and produce medium to large effect sizes at post-treatment, with a maintenance of effects at follow-up^12^. As a result, iCBT has established itself as a viable mode of treatment for depression and anxiety. Still, most research has explored iCBT’s efficacy under more controlled settings, with effectiveness trials in routine care finding mixed outcomes^12,13^. Most effectiveness trials were situated in specialized iCBT clinics. Research to assess iCBT’s effectiveness in non-specialized settings is scarce. In the UK, iCBT has previously been investigated within a primary care context (the REEACT trials)^13,14^. Outcomes from these trials were discouraging, finding iCBT to be neither more effective nor cost-effective than treatment as usual by general practitioners alone. Implementation challenges resulting in low up-take and usage of iCBT and less than optimal support provided during treatment (i.e. only minimal or non-therapeutic support when research emphasises the superiority of supported-iCBT)^15^ likely affected the trials’ outcomes.

We sought to evaluate the effectiveness and cost-effectiveness of iCBT for depression and anxiety in a pragmatic clinical trial within IAPT routine stepped care. We hypothesized that iCBT would reduce anxiety and depression severity over an 8-week treatment period compared to waiting-list control and be considered cost-effective based on criteria set out by the National Institute for Health and Care Excellence (NICE; UK). Effectiveness and cost-effectiveness were hypothesized to be maintained throughout follow-up.

## Results

### Baseline characteristics

**Fig. 1:**
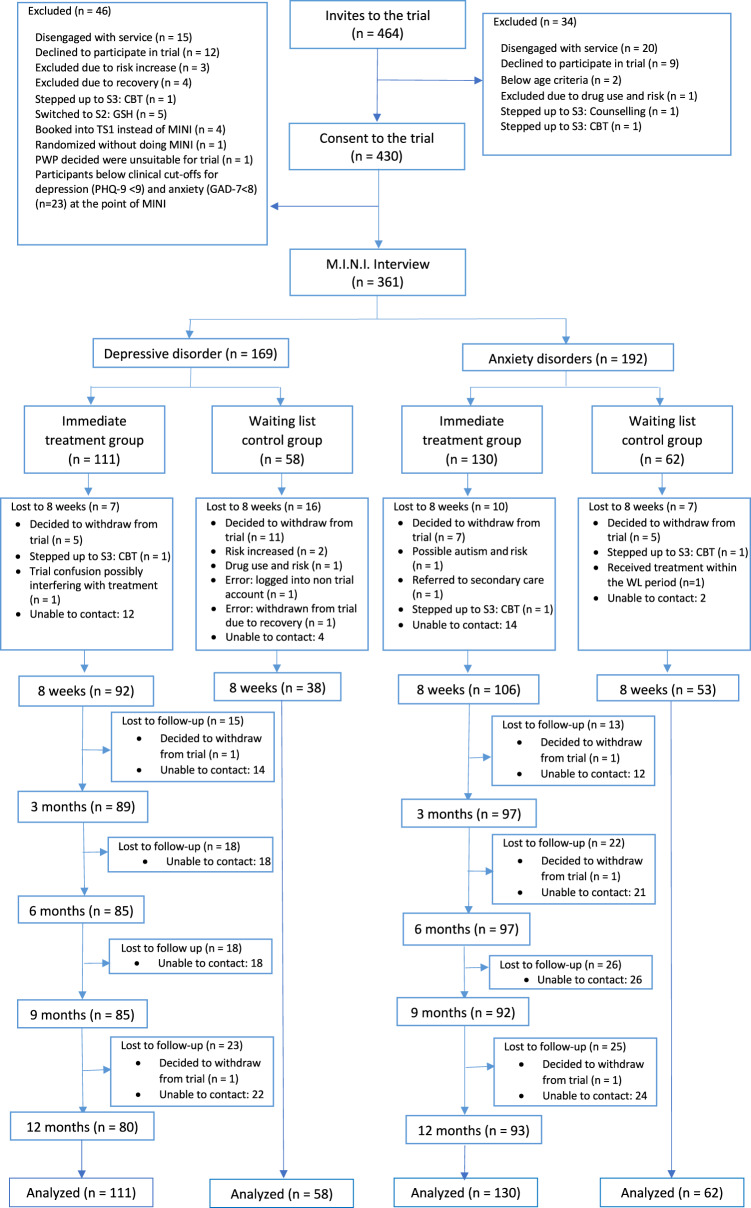
Flowchart of participants – CONSORT. CBT Cognitive Behavioural Therapy; GSH Guided Self-Help; MINI Mini International Neuropsychiatric Interview 7.0.2 (M.I.N.I.7.0.2); PHQ-9 Patient-Health Questionnaire-9; GAD-7 Generalised Anxiety Disorder-7; S2 Step 2 IAPT; S3 step 3 IAPT; PWP Psychological Wellbeing Practitioners; TS1 Telephone Screening 1.

Between 28 June 28 2017 and 30 April 2018, 464 participants were invited to the trial. Of those, 430 consented to the trial and 361 (84%) met all inclusion criteria and were randomized. Retention of participants for research purposes was highest at 8-weeks (intervention-arm: 82%; control-arm: 76%) and lowest at 12-months (intervention-arm: 72%; Fig. 1). The median age of participants was 29 (IRQ = 18) and 70% (258/361) were female (Table 1). At baseline, 80% (290/361) met criteria for at least one M.I.N.I.7.0.2 diagnosis and 70% (252/361) were at ‘caseness’ (defined thresholds as set by IAPT being PHQ-9 ≥ 10 and/or GAD7 ≥ 8) on both PHQ-9 and GAD-7 (Table 1).

**Table 1:** Baseline characteristics of study sample.

	No. (%)			Difference statistics and *p* values
	Overall (*N* = 361)	Intervention-arm (*N* = 241)	Waitlist control-arm (*N* = 120)	
Age, Median (IQR)	29 (18)	29 (19)	31 (18)	*U* = 13273; *p* = 0.20
Female sex	258 (70.6%)	173 (71.8%)	85 (70.8%)	*χ*^2^ = 0.04; *p* = 0.90
White ethnicity	304 (84.2%)	206 (85.5%)	98 (81.7%)	*χ*^2^ = 15.07; *p* = 0.52
Religion				
No religious affiliation	221 (63.0%)	156 (66.7%)	65 (55.6%)	*χ*^2^ = 10.64; *p* = 0.22
Christian	88 (25.1%)	54 (23.1%)	34 (29.1%)	
Other	52 (14.4%)	31 (12.9%)	21 (17.5%)	
Sexual orientation				
Heterosexual	307 (90.3%)	209 (92.1%)	98 (86.7%)	*χ*^2^ = 4.41; *p* = 0.35
Lesbian, gay or bisexual	26 (7.6%)	15 (6.6%)	11 (9.7%)	
Other	7 (2.1%)	3 (1.3%)	4 (3.5%)	
Employed	269 (74.5%)	184 (76.3%)	85 (70.8%)	*χ*^2^ = 1.28; *p* = 0.30
Comorbid LTC	61 (17.1%)	41 (17.3%)	20 (16.7%)	*χ*^2^ = 0.01; *p* = 0.93
Taking psychotropic medication	158 (43.8%)	99 (41.1%)	59 (49.2%)	*χ*^2^ = 2.04; *p* = 0.18
M.I.N.I.7.0.2 diagnosis	290 (80.3%)	193 (80.1%)	97 (80.8%)	*χ*^2^ = 0.03; *p* = 0.89
Major depressive disorder	189 (52.4%)	125 (51.9%)	64 (53.3%)	*χ*^2^ = 0.07; *p* = 0.82
Anxiety disorder(s)	231 (64.0%)	156 (64.7%)	75 (62.5%)	*χ*^2^ = 0.17; *p* = 0.73
Generalized anxiety disorder	199 (55.1%)	134 (55.6%)	65 (54.2%)	*χ*^2^ = 0.07; *p* = 0.82
Social anxiety disorder	63 (17.5%)	42 (17.4%)	21 (17.5%)	*χ*^2^ = 0.00; *p* = 0.99
Panic disorder	61 (16.9%)	42 (17.4%)	19 (15.8%)	*χ*^2^ = 0.14; *p* = 0.70
Comorbid depressive and anxiety disorder	130 (36.0%)	88 (36.5%)	42 (35.0%)	*χ*^2^ = 0.08; *p* = 0.82
PHQ-9, Mean (SD)	14.3 (5.0)	14.4 (4.9)	14.2 (5.1)	*t*(359) = 0.40; *p* = 0.68
GAD-7, Mean (SD)	12.6 (4.5)	12.7 (4.7)	12.5 (4.2)	*t*(359) = 0.24; *p* = 0.80
WSAS, Mean (SD)	18.0 (7.4)	17.4 (7.2)	19.4 (7.8)	*t*(359) = −2.48; *p* = 0.01

*LTC* long-term condition, M.I.N.I.7.0.2 Mini International Neuropsychiatric Interview, *PHQ-9* Patient Health Questionnaire, *GAD-7* Generalised Anxiety Questionnaire, *WSAS* Work and Social Adjustment scale.

### Effectiveness outcomes

#### Outcomes at post-treatment

**Fig. 2:**
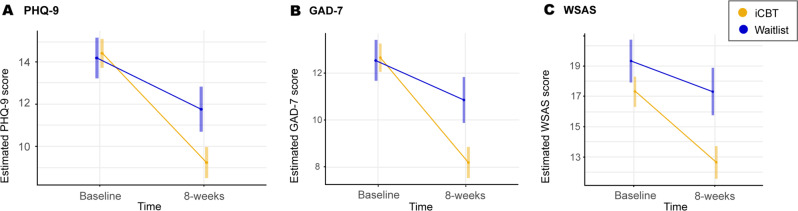
Estimated marginal means and confidence intervals (CI) for time by treatment group interaction effects at 8-weeks. **a** Random intercept linear mixed model suggesting significant time by intervention-arm interaction effect. Bonferroni adjusted estimated mean difference between intervention-arms at 8-weeks 2.52 (95% CI 0.78, 4.27, *p* = 0.0009). Between group effect size *d* = 0.55 (95% CI 0.32, 0.77). **b** Random intercept linear mixed model suggesting significant time by intervention-arm interaction effect. Bonferroni adjusted estimated mean difference between intervention-arms at 8-weeks 2.67 (95% CI 1.07, 4.26, *p* = 0.0001). Between group effect size *d* = 0.63 (95% CI 0.40, 0.85). **c** Random intercept linear mixed model suggesting significant time by intervention-arm interaction effect. Bonferroni adjusted estimated mean difference between intervention-arms at 8-weeks 4.68 (95% CI 2.11, 7.24, *p* < 0.0001). Between group effect size *d* = 0.35 (95% CI 0.13, 0.57).

In evaluating primary study outcomes, 8-week models suggested significant interaction effects of time-by-intervention-arm for PHQ-9 (*b* = −2.75; *SE* = 0.64; 95% CI −4.00, −1.50; *p* < 0.0001; observed power 0.99) and GAD-7 (*b* = −2.79; *SE* = 0.61; 95% CI −4.00, −1.58; *p* < 0.0001; observed power 0.99). Paired comparisons indicated that in those who received iCBT, depression and anxiety symptoms were reduced from baseline to 8-weeks more than in those who did not (Fig. 2 and Supplementary Table 1 for further details). Table 2 shows observed and estimated means for each group and timepoint. Utilizing the same model as above, there was a significant time-by-intervention-arm effect for the WSAS (*b* = −2.65, *SE* = 0.99, 95% CI −4.59, −0.72; *p* = 0.075; observed power 0.77) and significantly lower functional impairment in the intervention-arm at 8 weeks.

**Table 2:** Observed and estimated marginal means across time-points and by treatment group.

	Treatment group					Waitlist control group				
	Observed			Estimated		Observed			Estimated	
	*N*	Mean	SD	Mean	SE	*N*	Mean	SD	Mean	SE
Baseline										
PHQ-9	241	14.41	4.94	14.41	0.35	120	14.18	5.11	14.18	0.49
GAD-7	241	12.66	4.69	12.66	0.31	120	12.54	4.18	12.54	0.44
WSAS	241	17.35	7.15	17.30	0.50	120	19.38	7.81	19.30	0.71
8-weeks										
PHQ-9	198	9.28	5.94	9.24	0.37	91	11.58	5.67	11.76	0.54
GAD-7	198	8.2	5.31	8.18	0.34	91	10.79	5.12	10.85	0.49
WSAS	198	12.71	8.31	12.60	0.54	91	16.89	8.60	17.30	0.80
3-month										
PHQ-9	186	8.17	5.69	8.76	0.41					
GAD-7	186	7.38	5.32	7.95	0.38					
WSAS	186	11.77	8.29	12.22	0.57					
6-months										
PHQ-9	182	7.16	5.82	7.71	0.40					
GAD-7	182	6.93	5.52	7.35	0.38					
WSAS	182	10.34	8.77	10.91	0.59					
9-months										
PHQ-9	177	6.81	5.71	7.07	0.41					
GAD-7	176	6.48	5.14	6.72	0.37					
WSAS	177	9.98	8.67	10.23	0.61					
12-months										
PHQ-9	173	6.79	5.54	6.62	0.40					
GAD-7	173	6.08	4.81	6.01	0.36					
WSAS	173	10.01	8.54	9.87	0.61					

Estimated marginal means in the intervention-arm at 8-weeks are based on 8-week linear mixed models. Estimated means at 3-, 6-, 9-, and 12-months follow-up are based on follow-up marginal models.

### Follow-up outcomes

**Fig. 3:**
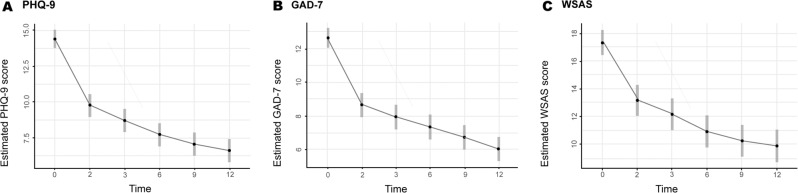
Intervention-arm estimated marginal means and confidence intervals (CI) across all time-points (in months). **a** Marginal linear model showing estimated mean PHQ-9 score reductions across all timepoints. Bonferroni adjusted estimated mean difference between 8-weeks and (i.e. 2 months) and 12-months 3.12; SE = 0.44; 95% CI 1.82, 4.39; *p* < 0.0001). **b** Marginal linear model showing estimated mean PHQ-9 score reductions across all timepoints. Bonferroni adjusted estimated mean difference between 8-weeks and (i.e. 2 months) and 12-months 2.60; SE = 0.40; 95% CI 1.43, 3.76; *p* < 0.0001). **c** Marginal linear model showing estimated mean PHQ-9 score reductions across all timepoints. Bonferroni adjusted estimated mean difference between 8-weeks and (i.e. 2 months) and 12-months 3.24; SE = 0.62; 95% CI 1.40, 5.07; *p* < 0.0001).

Follow-up models demonstrated maintained or improved symptom levels across all follow-up time-points (Fig. 3). Paired comparisons confirmed significant improvements from 8-weeks to 12-months on PHQ-9 (mean difference 3.12; SE = 0.44; 95% CI 1.82, 4.39; *p* < 0.0001), GAD-7 (mean difference 2.60; SE = 0.40; 95% CI 1.43, 3.76; *p* < 0.0001) and WSAS (mean difference 3.24; SE = 0.62; 95% CI 1.40, 5.07; *p* < 0.0001; see Supplementary Table 2 for model coefficients and Table 2 for observed and estimated means across measures).

### Clinically significant change

At post-treatment (8-week), 46.4% (90/194) of the intervention-arm relative to 16.7% (15/90) control-arm participants recovered, with 63.4% (123/194) intervention-arm relative to 34.4% (31/90) control-arm participants showing reliable improvement. Reliable recovery in the intervention-arm was 40.7% (79/194), and 13.3% (12/90) in the control arm. All between-group differences were significant (*p* < 0.01). Amongst 3-month M.I.N.I.7.0.2 completers (*n* = 179), 60% (18/30) with depression, 50% (24/48) with anxiety, and 46% (30/65) with comorbid depression and anxiety diagnoses, did not meet diagnostic criteria anymore at 3-months. Overall, 56.4% (101/179) of participants did not meet diagnostic criteria for any disorder at 3-months (see Supplementary Tables 3 and 4 for details).

### Platform usage

In terms of usage of the platform by 8-weeks, participants had on average 13.1 logins (SD = 10.8, median = 11, IQR = 12), used an activity 94.2 times (SD = 90.1, median = 68, IQR = 115 25), spent 3 h 58 min on the platform (SD = 219 min, median = 204 min, IQR = 220 5 min) and completed 41% of the programme (SD = 0.24, median 38%, IQR = 36%). By 12 months, the average usage increased up to 22.3 logins (SD = 17.9, median 18, IQR = 17.75), used an activity 126.1 times (SD = 133.3, median = 98, IQR: 130.75), 5 h 55 min spent (SD = 336 min, median = 295 min, IQR: 323.53 min) and 56% of the programme completed (SD = 0.29, median 61%, IQR = 48%). Participants received an average of 4.7 online reviews (SD = 2.4, median = 5, IQR = 2.75).

### Cost-effectiveness

In terms of preference-based health status, the EQ-5D-5L tariff score in the ITT analysis suggest a sustained health improvement over 12 months relative to baseline for those using iCBT, and at a higher magnitude relative to control at 8-weeks (Supplementary Tables 5 and 6). To give an indication of reported resource-use and associated costs, descriptive statistics (i.e. response rates, resource-use, and associated costs) by trial-arm in both, Complete-Case and ITT, analyses are presented in Supplementary Tables 7–9.

**Fig. 4:**
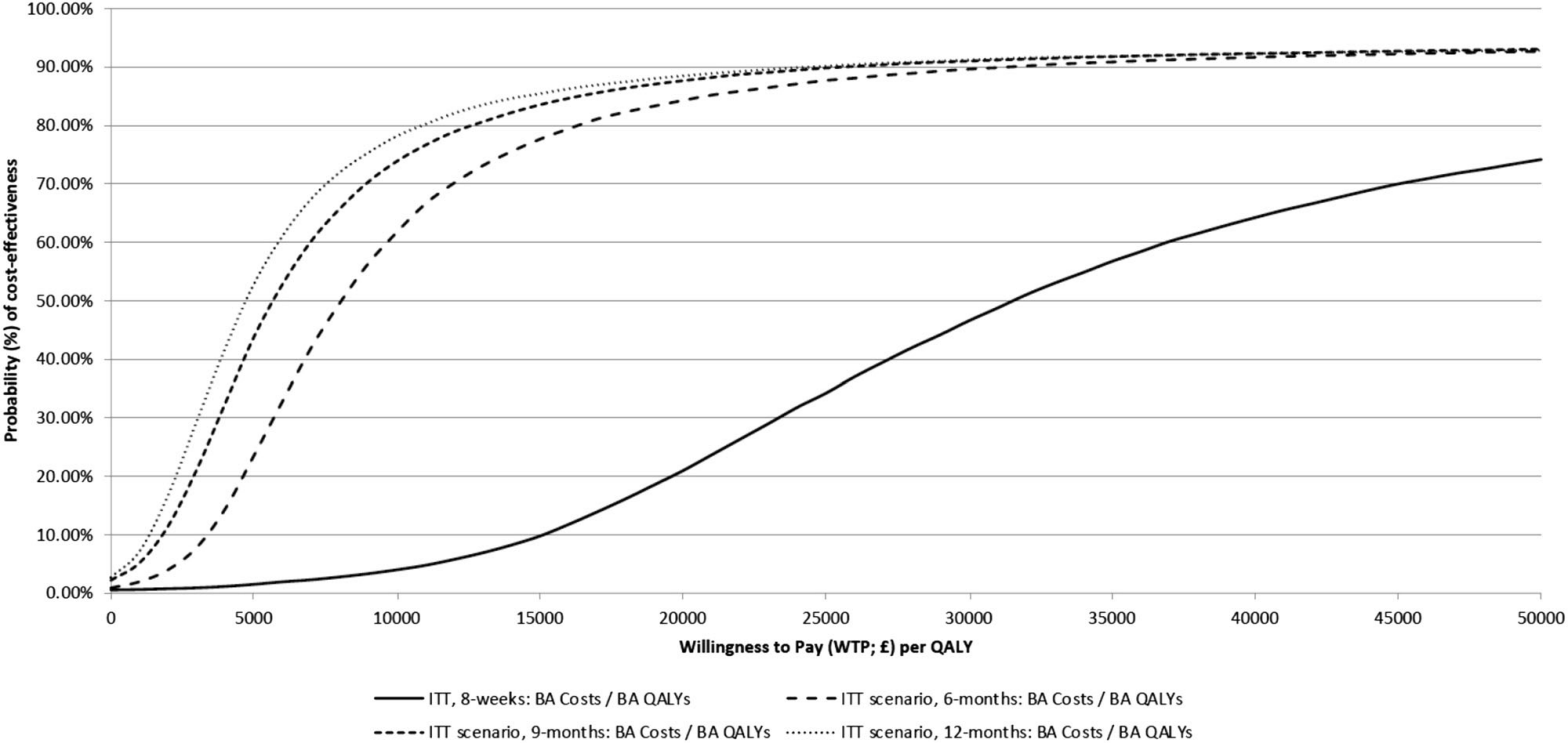
CEAC representing probability of cost-effectiveness of iCBT relative to waiting-list control over 8 weeks, and predicted over 6, 9, and 12 months. ITT Intention-to-treat, BA Baseline-Adjusted; QALY Quality-Adjusted Life year.

The incremental CEA and within trial-group results are presented in Supplementary Tables 10 and 11. Figure 4 presents the cost-effectiveness acceptability curve suggesting probability of iCBT cost-effectiveness dependent on willingness-to-pay per QALY, by time-horizon of the baseline-adjusted analyses. The base-case CEA (baseline-adjusted costs/baseline-adjusted QALYs) suggests over 8-weeks, iCBT has a high probability of producing QALY gains (>96.6%), but low probability of cost-savings (<0.5%), at an estimated iCBT intervention cost of £94.63 per person (Supplementary Table 12) against the no-intervention-cost waiting-list control. Across base-case analyses, probability of cost-effectiveness at NICE’s upper £30,000 per QALY threshold ranged from 46.6% (baseline-adjusted QALY and baseline-adjusted costs ITT analysis; ICER = £29,764) to 65.5% (unadjusted ITT analysis; ICER = £20,310); however, these probabilities decrease as the willingness-to-pay threshold decreases e.g. 21.0–47.9% within the same aforementioned analyses at NICE’s lower £20,000 per QALY threshold over 8-weeks.

In the scenario analyses at 6, 9 and 12 months, the probability of cost-effectiveness increases as the time horizon increases (Fig. 4). The probability of cost-effectiveness at 12-months ranges from 91.2% to 92.0% at £30,000, or 88.5–90.1% at £20,000, per QALY gained dependent on analysis conducted (Fig. 4 only shows baseline-adjusted costs/baseline-adjusted QALYs). An interpretation of these scenario analyses relative to base-case results is provided in the Supplementary (section 4).

### Adverse effects

Adverse effects were rare. Amongst 8-week measure completers, 5.2% (10/194) in the intervention-arm and 12.2% (11/90) in the waiting-list deteriorated (i.e. increases of PHQ-9 ≥ 6 and/or GAD-7 ≥ 4). Amongst those who completed the M.I.N.I.7.0.2 at 3 months, 4.47% (8/179) moved from subthreshold symptoms of anxiety and/or depression at baseline to a clinical diagnosis at 3 months. No severe adverse events were reported. During follow-up, 55/241 intervention-arm participants (25.70%) either self-reported or were recorded by IAPT to have received further mental health treatment (Supplementary Table 13).

## Discussion

This large-scale RCT conducted within IAPT showed that iCBT for depression and anxiety is effective as a standalone intervention when fully integrated and operated in non-specialised routine stepped-care settings. The pragmatic trial design increases the ecological validity of the observed effectiveness of iCBT for depression and anxiety. The primary hypothesis of the study was confirmed by showing iCBT to be effective at the end of the 8-week intervention period compared to waiting-list control, with further statistically significant improvements in the primary outcomes over the 12-month follow-up. The probability of cost-effectiveness was predicted to be much higher over longer time-horizons than that observed over the 8-week treatment period alone, driven by QALY-based health improvement, which in part aligns with our clinical and longer-term cost-effectiveness hypotheses.

The results build upon prior effectiveness evidence of supported iCBT interventions for individuals with depression and anxiety disorders in primary care^12,15^. The observed effects also align with previous reviews in terms of the type of comparator, where waitlists comparators lead to larger effects that when compared to treatment as usual^12^. In the context of UK’s primary care, the observed effects outperform the findings of the REEACT trials^13,14^, which could be explained by the stepped-care model within which these interventions were embedded, with contextual factors favouring the deployment and uptake of the interventions (i.e. trained personnel for the use of iCBT, routine outcome monitoring). The significant improvements on depression and anxiety symptoms observed in the follow-up compared to the end of treatment period have also been shown in prior reviews^12,15^. In addition, the statistically significant improvement in functional impairment is coherent with the intervention’s effects on depression and anxiety^16^.

Regarding clinical diagnoses, in the current study between 46% and 60% of individuals did not meet diagnostic criteria anymore for one or multiple diagnoses after receiving the iCBT intervention, which is comparable to rates observed in previous studies of iCBT^17^. In this regard, the rates of recovery from diagnosis align with the rates of reliable improvement and recovery as assessed using the primary outcomes. It is important to consider that in our calculations of clinical improvement, we did not rely on the continuous assessments from the IAPT routine outcome monitoring system but we used different methods to account for missing data^5^. Therefore, our outcomes are not directly comparable to publicly reported IAPT outcomes as they are calculated differently.

Over 8 weeks, the baseline-adjusted ITT cost-effectiveness result suggests an ICER of £29,764 which is below the £30,000 per QALY NICE ICER upper threshold. This produced a probability of cost-effectiveness of 46.6% due to the skewed nature of the estimates driven by a high probability of QALY gains, but low probability of cost-savings. Short comparative trial time-horizons, which are often associated with waiting-list designs due to ethical reasons when with-holding care for longer-periods, limit potential QALY gains which are time dependent (i.e. more potential for QALY gains over 12 months relative to 8 weeks). Thus, a statistical extrapolation of costs and outcomes in the control were used to predict potential cost-effectiveness beyond 8-weeks, which suggested much higher probabilities of cost-effectiveness (i.e. >91% at £30,000 per QALY over 12 months) driven by the sustained health improvement observed in the intervention-arm than predicted in the control. These findings support the potential for internet-delivered interventions to be a cost-effective complement to established interventions when assessing cost-effectiveness beyond the 8-week treatment period^11^. Decision makers wanting to use the results from the scenario analyses based on the statistical extrapolation should first consult the extended discussion and exploration of the scenario analyses and what these results represent in Supplementary (section 4). The results align with previous findings from economic evaluations of e-health interventions in different settings, which support the potential of iCBT to be a cost-effective complement to established interventions^11,18^.

Although there was a low estimated probability of cost-savings from a health and social care perspective, this aligns with evidence from previous studies. Effective treatments of depression (in particular) tend to show an increase in ‘direct’ costs (e.g. intervention, hospital, primary care), with cost-savings often associated with decreased ‘indirect’ costs (e.g. lost productivity from absenteeism and presenteeism)^19^. Our costing perspective (as per the NICE reference case) does not account for wider societal costs and so does not capture these cost aspects.

Participants use of the interventions were similar to that observed in a previous trial of the same platform (i.e. around 5 h of usage and 14.7 logins), which also produced positive outcomes compared to a waiting-list group^16^. These results align with previous literature of iCBT, where it has been found that users do not need to complete all the intervention to benefit from it^20,21^. In this regard, there should be a shift in how adherence, and implicitly attrition, to iCBT is conceptualised, from a more traditional conceptualisation of completing all the modules, towards determining how much exposure is enough to produce clinical benefits^20^.

The execution of the trial within an IAPT service where iCBT is currently used as per normal service procedures is a key strength of the study, since it increases the ecological validity of the findings, by utilizing a real-world context^22^. In addition, the service population where we undertook the study has socio-demographic and clinical characteristics which are representative of the overall IAPT population, although female gender was slightly over represented compared to IAPT reports (70% females in this trial vs. 65% in IAPT referrals)^8^. The addition of clinician-administered psychiatric interviews, alongside the self-reported clinical assessments (i.e. PHQ-9, GAD-7, WSAS) increases the study’s clinical validity. The assessment of cost-effectiveness of iCBT within a stepped-care model (i.e. IAPT), despite the observed CEA being limited to 8-weeks is another strength of the study.

This study also has several limitations. First, the impossibility of following-up the waiting-list group for ethical reasons prevents observed analysis of treatment effects and cost-effectiveness results beyond 8-weeks; however, the maintained iCBT treatment effect (relative to baseline) and scenario CEA suggests promising, positive long-term outcomes (see also Supplementary section 4, for an extended discussion around the scenario analyses). Second, a waiting-list comparator was used instead of care as usual or an active comparator, which would be more reflective of what usually would happen in the absence of this iCBT intervention. Furthermore, the use of a WL group might also produce an overestimation of the treatment effects, since research has shown larger effects sizes in favour of this type of control group when compared to treatment as usual comparators^12^. Despite being common in anxiety research on psychological interventions, there are also other inherent issues with wait-list comparators such as issues of expectancy^23^.

Third, inter-rater reliability for the M.I.N.I.7.0.2. was not assessed in this study which calls into question the internal validity of the results, although the high coefficients observed in validation studies of the M.I.N.I. and the training and supervision provided to the PWPs who were involved ensured that they properly administered the interview. Fourth, asking participants to self-report resource-use over the past three months using the CSRI might have produced recall errors and subsequent recall-bias. Fifth, the fact that the trial was conducted within routine care settings caused that some users were still in treatment by the end of the 8-week period, which could have influenced the effects of the intervention at this timepoint. Sixth, although support was stated on the protocol to be offered online, some reviews were conducted over the phone (as per service-related reasons). Future secondary analyses will explore potential confounding effects of telephone reviews further. Finally, the study was powered to detect between-groups differences based on F-tests; however, after powering the study but before trial completion and unblinding of the data, it was decided to apply linear mixed models, since they are more robust analyses (see “Supplementary Methods”). Post-hoc power analyses for the primary outcomes indicated power to have been in excess of the assumed 80% and therefore adequate in detecting moderate between-groups effect sizes for the primary outcomes.

Limitations related to the CEA include that the predicted costs and effects beyond 8-weeks for waiting-list control account for the intervention’s treatment effect, which potentially underestimates the QALYs gained and cost-differences between trial-arms. Trial comparisons against waiting-list controls tend to estimate higher intervention-related QALY gains than against ‘active’ controls (e.g. face-to-face CBT), which limits the findings of this study compared to other active interventions (e.g. other delivery methods of CBT). Increased productivity could well be a source of cost-savings from this iCBT intervention which can’t, or are unlikely to, be observed in our CEA given the costing perspective and short trial time-horizon. We applied a mean intervention cost to all participants, rather than costing the intervention to the specific participant, due to the inability to retrospectively link specific PWPs to trial participants which will have an effect on how the uncertainty around the intervention cost is accounted for in the bootstrap replicates and subsequent CEAC.

Given that we compared iCBT to a waiting-list group in IAPT, it would be valuable for future studies to examine the relative efficacy of iCBT compared to other low-intensity interventions offered within IAPT (i.e. guided self-help, psychoeducational groups). Furthermore, a trial analysing effectiveness and cost-effectiveness of iCBT compared to face to face CBT would shed more light into the potential of iCBT to produce cost-savings and thus make further recommendations on service funding and delivery. To complement quantitative findings, we included qualitative interviews examining the facilitators and barriers that affected the implementation of the current intervention in IAPT services and these will be published separately. Distinct support models (for example support-on-demand) could shed light on the role of the supporter within a stepped-care model. Usage and engagement metrics, more easily measurable within an iCBT modality, can add to the understanding of the therapeutic process.

The results of the current study, conducted within a routine, NHS IAPT setting, show statistically significant and sustained reduction in depression and anxiety severity which translated into QALY-based health improvements, considered cost-effective by criteria typically used by NICE (albeit against a waiting-list control) when assessed beyond the 8-week treatment period. These results consolidate iCBT for depression and anxiety as an effective treatment alternative within stepped-care models. The results of this trial support the findings observed in trials from other countries where iCBT was implemented and assessed as part of stepped care and collaborative care models^24,25^. In a society where the prevalence for depression and anxiety is rising and demand is outpacing what mental health services are able to offer, embedding digital interventions as treatment alternatives increases accessibility and service efficiency, which has the potential to be cost-effective for the health care system.

## Method

### Study design

An 8-week randomized controlled trial (RCT) with 2:1 (iCBT intervention: waiting-list control) allocation, and 12-month follow-up in the intervention-arm. This study uses a pragmatic design where the trial procedures were built around the standard service procedures used at step 2 of IAPT and the support was offered by service employees. The study setting was an NHS IAPT Trust in England (i.e., a local health service provider covering the population within a geographical area). At Step 2 IAPT offers low-intensity psychological interventions to individuals experiencing mild to moderate symptoms of depression and anxiety. The trial protocol^26^ was approved by the National Health Service (NHS) England Research Ethics Committee (REC Reference: 17/NW/0311). The trial was prospectively registered: Current Controlled Trials ISRCTN91967124; Clinicaltrials.gov Identifier: NCT03188575. See “Supplementary Methods” for declarations of protocol deviations and measures reported.

### Participants

Participants were chosen based on IAPT standard operating procedures to best represent participants who may be eligible and require iCBT as part of usual IAPT care. Participants were new IAPT referrals, included if aged between 18–80 years, screened for psychological pathology using clinical thresholds for Patient-Health Questionnaire-9 (PHQ-9 ≥ 9)^27^ and/or Generalised Anxiety Disorder-7 (GAD-7 ≥ 8)^28^, and suitable for iCBT (i.e. willing to engage in iCBT, internet access). Exclusion criteria included: suicidal ideation/intended (PHQ-9 question 9 score > 2 and/or expressed during clinical interview); psychotic illness; organic mental health disorder; alcohol and/or drug misuse; and currently receiving psychological treatment. Recruited participants signed informed consent via digital signature.

### Randomization and masking

Participants were randomized at an individual level using an algorithm developed by a computer scientist (Sealed Envelope, 2016) and executed independently of the research team, employing random permuted blocks using block sizes of 9, and including stratification within a 2:1 allocation ratio between treatment and waiting list control groups. The Psychological Wellbeing Practitioners (PWP) who carried out the support and assessment of the patients could not be blinded to allocation for practical reasons.

### Procedures

As per routine practice, clients attending the IAPT service received an assessment of their needs which determined their initial presentation (depression/anxiety), suitability for Step 2 and for iCBT, and allocation to one of the available programmes. Subsequently, eligible participants were invited to the trial and thereafter, as part of this research trial, signed informed consent via digital signature, completed the baseline research assessments and completed the M.I.N.I.7.0.2 with a PWP to determine a primary diagnosis of depression or anxiety with initial treatment assignment as per routine service’s practice (most suitable intervention based on their symptomology). Given the higher prevalence of anxiety disorders compared to depression^29^, primary diagnosis was used for randomization to ensure a balanced sample; this data will inform future secondary analyses. Those randomized to the intervention-arm commenced their treatment with their PWP supporter.

The iCBT programs were SilverCloud Health’s ‘*Space from Depression’*, ‘*Space from Anxiety’* and ‘*Space from Depression and Anxiety’* interventions, whose efficacy has been previously supported^16^ and which have been described in detail elsewhere^26^. The interventions share core CBT content with customisations for depression and specific anxiety-disorder presentations (e.g., social anxiety, generalized anxiety, panic disorder, etc.). Participants were supported during their iCBT treatment by PWPs, who are psychology graduates specifically trained in the provision of low-intensity CBT interventions^5^. PWPs were located in offices across several localities in the area, which are separate to primary care premises. Assessment and triage for services typically occurs over the phone but can sometimes occur in face-to-face settings. PWPs assessed participants for suitability, assigned participants to a specific iCBT programme, monitored their progress throughout the trial and provided regular reviews. During the reviews, PWPs provided feedback to clients based on their work from week-to-week (e.g. modules completed, tools used, shared journal entries) and encouragement in order to promote meaningful engagement with the programme^30^. PWPs were instructed to provide six online reviews (15 min per participant per review) over the 8-week intervention period. Potential risk to participants was monitored in line with routine service procedures (see protocol for full description of risk management). Fidelity to the treatment was ensured through checklists and clinical supervision offered by case managers. Specifically, the checklist assessed if the content of the review offered by the supporter considered the client, the tools used and the previous levels of engagement of the client when writing the review. In total, 176 checklists were completed that represented 85 cases with an average of 2 supervisions per case. One-hundred-and-seventy (*n* = 170) of the checklists were marked as compliant with the content of online reviews. 6 cases were either not indicated as compliant (reason not stated) or marked as the client not attending the session.

After 8 weeks, all participants were asked to complete relevant measures online and the control-arm participants began treatment. Follow-up procedures included telephone administration of the Mini International Neuropsychiatric Interview 7.0.2 (M.I.N.I.7.0.2)^31^ at 3-month; the timeframe was decided in order to allow enough time for the intervention to have an effect (8 weeks), plus one month that is required by diagnostic criteria for some disorders (e.g. panic disorder, social anxiety disorder) to be asymptomatic in order to meet the criteria. Thereafter, online self-reported measures were administered at 3-, 6-, 9-, and 12-month follow-up timepoints. In order to enhance retention of participants for research purposes only, participants received financial incentives in the form of vouchers (up to £97.50) for the completion of research measures (“Supplementary Methods”). For trial purposes, PWPs were formally trained in the procedures they would be undertaking that were outside of normal service (i.e. diagnostic interview, checking for consent, monitoring fidelity).

### Outcomes

The primary outcome measures were the PHQ-9 for depressive symptoms^27^ and the GAD-7 for anxiety symptoms^28^.

Secondary outcomes included rates of ‘significant improvement’ and ‘recovery’, based on PHQ-9 and GAD-7 scores at 8-weeks. These were calculated as follows: (a) significant improvement: a score change of PHQ-9 ≥ 6 and/or GAD-7 ≥ 4, where the thresholds are calculated based on Jacobson’s & Truax’s criteria for clinically significant change, provided they do not reliably deteriorate on either measure (b) recovery: moving from ‘caseness’ (defined thresholds as set by IAPT being PHQ-9 ≥ 10 and/or GAD7 ≥ 8) to ‘non-caseness’ (below the threshold on both measures)^32^. The Work and Social Adjustment Scale (WSAS) was used to measure functional impairment levels^33^. The M.I.N.I.7.0.2 was administered to determine diagnosis of depression and/or anxiety disorder at baseline and 3-months follow-up.

Rates of deterioration at post-treatment (increase in PHQ-9 ≥ 6 and/or GAD-7 ≥ 4) and an increase in the number of diagnoses at 3-months were considered as adverse events. Metrics of programme usage such as time spent in the platform, number of logins and percentage of programme completion were collected for those participants assigned to the iCBT group.

Cost-effectiveness analysis was based on the preference-based EuroQoL Five-Dimension Five-Level (EQ-5D-5L)^34^ health status measure using the EQ-5D-5L cross-walk algorithm as NICE’s interim-position^34,35^ for ‘cost per quality-adjusted life year (QALY)’ analysis. An adapted Client Service Receipt Inventory (CSRI; routinely completed at study site, see supplementary Fig. 1)^36^ was used to collect self-reported care resource-use over the past 3 months (note, the CSRI at 8-weeks asked about resource-use over the past 3 months, rather than previous 8 weeks as intended, and was not administered at 3-month follow-up).

Measures were administered at baseline and 8-weeks forming the primary endpoint between trial-arms, with further intervention-arm follow-up at 3-, 6-, 9-, and 12-month. At baseline, demographic details were collected.

### Statistical analysis

Sample size was determined separately for primary presentations of depression and anxiety. In total, 360 participants were required to detect a moderate between-group effect size (*d* = 0.5) on repeated-measures for PHQ-9 and GAD-7, based on F-tests, with a two-tailed α of .05, power of 80%, and 2:1 randomisation procedure (to reduce likelihood of having many people waiting). The expected between-group difference and an aggregated 25% uplift to ameliorate against attrition were based on a previous study and a meta-analysis of iCBT for depression^16,37^. Due to a discrepancy between the original formula for statistical power, based on F-tests, and the statistical analysis actually applied (i.e. linear mixed models; “Supplementary Methods”), statistical power of the mixed effects models was checked through the R package nlmeU and it was found adequate.

Baseline demographic and outcome variable differences between groups, and measure responders versus non-responders, were investigated through Chi-squared, Mann-Whitney and t-tests. Missing data mechanisms were assessed separately at 8-weeks (across and within trial-arms) and throughout follow-up (intervention-arm only), using Little’s ‘missing completely at random’ (MCAR) test. Missing data was deemed to be ‘MCAR’ at 8-weeks (see Supplementary Table 3). Across follow-up, data was deemed to be ‘missing at random (MAR)’ (see Supplementary Table 4).

Linear mixed and marginal models were used to evaluate intervention effectiveness, both considered robust intention-to-treat (ITT) analyses. Analyses included all available data from all participants. Graphical and statistical checks were conducted to assess model assumptions. These checks did not flag any major violations of assumptions. Six models were built, one up to 8-weeks across arms (‘8-week model’) and one including all intervention-arm data (‘follow-up model’) per outcome variable (PHQ-9, GAD-7, and WSAS). 8-week models were random intercept linear mixed models and included maximum likelihood estimation. Follow-up models were marginal models with an unstructured correlation structure as these produced superior model fit than corresponding linear mixed models. Given marginal model assumptions and MAR data at follow-up, follow-up analyses were preceded by multiple-imputation via multilevel joint modelling^38,39^. Bonferroni adjusted paired comparisons based on estimated marginal means were conducted to explore change between time-points and differences between the intervention-arms, and any subgroups. Between-group Cohen’s *d* effect sizes were calculated utilising raw score standard deviations^40^. Analyses were conducted in IBM SPSS version 26 and R version 3.6.1 using the ‘nlme’, ‘nlmeU’, ‘mitml’, ‘geepack’ and ‘emmeans’ packages.

### Cost-effectiveness analysis

Cost-effectiveness analyses were based on NICE’s reference case^41^ from a health and social care perspective, with QALYs elicited using the area under the curve (AUC) method^42^ from the EQ-5D-5L cross-walk algorithm as NICE’s interim-position^34,35^. Application of unit costs for 2017/18 in Great British Pounds (GBP, £) to per patient resource-use to estimate intervention and downstream costs is described in Supplementary Tables 14 and 15.

The CEA were conducted on a complete-case (CC) and ITT basis, with missing cases imputed for the latter using multiple-imputation by chained equation (MICE) for MAR data^43^. Incremental mean-point estimates of mean cost differences (intervention minus control) over mean QALY differences between trial-groups was used to determine incremental cost-effectiveness ratios (ICERs). Baseline adjustments (BA) were made using ordinary least squares (OLS) regression models with covariates including trial-group and: (1) 3-months pre-baseline costs for costs; (2) baseline EQ-5D-5L cross-walk score for QALYs; with both unadjusted and adjusted estimates reported to aid transparency in the reported results^43^. Non-parametric bootstrapping was used to calculate bootstrapped 95% confidence intervals (bCIs) and standard errors (bSE) around costs and effects, and for plotting cost-effectiveness acceptability curves (CEACs). CEACs present the probability of intervention cost-effectiveness compared to control across a range of decision maker willingness-to-pay (WTP) thresholds e.g. NICE’s £20,000 to £30,000 per QALY thresholds^41^.

The base-case CEA is conducted over the initial 8-weeks. As part of scenario analyses, regression-based extrapolations were used to estimate QALYs and costs beyond 8-weeks for the waiting-list control. In the ITT dataset for the intervention-arm, OLS models were fitted to the (EQ-5D-5L) tariff score/(downstream) costs at 3 (not for costs), 6, 9 and 12 months as the response variable, independently, and tariff score/costs at baseline and 8 weeks as the explanatory variables. The predicted models were then fitted to the waiting-list control’s data to estimate their potential tariff score/costs at the aforementioned time-points from which total costs and QALYs could be estimated, and CEA conducted. A step-by-step guide describing the scenario analyses is provided in Supplementary (section 4). Analyses were conducted in Stata version 15.

### Data Monitoring and Trial Managing Committee

The Data Monitoring and Trial Managing Committee (DMTMC) was composed of the following personnel: the SilverCloud data manager and two trial managers, Berkshire NHS representatives including the PI and Co-PI at Berkshire, two R&D clinician research assistants, and two R&D research assistants. The DMTMC met weekly and minutes were recorded at each of these meetings. Each week participants progress was monitored for safety and routine clinical decisions e.g. stepping up a participant from low-intensity to high-intensity treatment were communicated to the DMTMC and recorded (see consort flow diagram). To ensure the validity and integrity of the data collected throughout the trial period, the data manager provided weekly progress reports to the DMTMC.

### Reporting summary

Further information on research design is available in the Nature Research Reporting Summary linked to this article.

## Supplementary information

Supplementary Information

Reporting Summary

## Data Availability

The dataset analysed during the current study is available upon request to the corresponding author.

## Code availability

The code used to generate the statistical outputs is available upon request to the corresponding author.
