# Author Correction: A pragmatic randomized waitlist-controlled effectiveness and cost-effectiveness trial of digital interventions for depression and anxiety

**DOI:** 10.1038/s41746-020-0298-3

**Published:** 2020-06-30

**Authors:** Derek Richards, Angel Enrique, Nora Eilert, Matthew Franklin, Jorge Palacios, Daniel Duffy, Caroline Earley, Judith Chapman, Grace Jell, Sarah Sollesse, Ladislav Timulak

**Affiliations:** 1 University of Dublin, Trinity College, School of Psychology, E-mental Health Research Group, Dublin, Ireland; 2 Clinical Research & Innovation, SilverCloud Health, Dublin, Ireland; 3 HEDS, ScHARR, University of Sheffield, Sheffield, England; 4 Berkshire Healthcare NHS Foundation Trust, London, Berkshire England

**Keywords:** Health services, Health care economics

Correction to: *npj Digital Medicine* 10.1038/s41746-020-0293-8, published online 15 June 2020

Following publication it was found that the affiliation information for author Ladislav Timulak was incorrect. The correct affiliation for Ladislav Timulak is as follows:

University of Dublin, Trinity College, School of Psychology, E-mental Health Research Group, Dublin, Ireland.

The affiliation information has been updated in both the HTML and PDF versions of the paper.

