# Supplementary material for: A pragmatic randomized waitlist-controlled effectiveness and cost-effectiveness trial of digital interventions for depression and anxiety
Source: NPJ Digit Med. 2020 Jun 15;3:85. doi: 10.1038/s41746-020-0293-8 (PMC7295750; doi:10.1038/s41746-020-0293-8)
Supplement: Supplementary file 1 — Supplementary Information [file 41746_2020_293_MOESM1_ESM.pdf]

Supplementary Material

Richards D., Enrique A, Eilert N, Franklin M, Palacios J, Duffy D, Earley C, Chapman J, Jell G, Sollesse S, and Timulak L. A pragmatic randomized waitlist-controlled effectiveness and cost-effectiveness trial of digital interventions for depression and anxiety.

Table of Contents

Results 2
1. Effectiveness analysis 2
2. M.I.N.I.7.0.2 outcomes 3
3. Cost-effectiveness analysis 3
3.1. EQ-5D-5L completion rates 3
3.2. EQ-5D-5L cross-walk tariff scores and subsequent QALYs by time-point and trial-arm 4
3.3. Downstream care resource-use and associated costs by trial-arm 5
3.4. Summary cost-effectiveness results by trial-arm and incremental results 7
3.5. Intervention cost 10
4. Further exploring the scenario analyses 10
5. Further treatment during follow-up period 14
Methods 16
Deviations from protocol 16
Declaration of reported measures 17
Cost-effectiveness analysis 17
Missing data analysis 20
Supplementary Figures 22

Results

1. Effectiveness analysis

All treatment period models included random intercepts, which suggested significant variability in PHQ-9, GAD-7 and WSAS scores at the level of the individual (PHQ-9 SD=3.93; 95% CI 3.35, 4.41; GAD-7 SD=3.31, 95% CI 2.91, 3.77; WSAS SD=5.66, 95% CI 5.22, 6.13). Fixed effect and interaction effect coefficients for the treatment period models can be found in Supplementary Table 1. Follow-up marginal linear models employed an unstructured correlation structure, which indicated correlations ranging from 0.25 to 0.72 between different time-points across models. Multiple imputation pooled model estimates and confidence intervals can be found in Supplementary Table 2. Observed and estimated PHQ-9, GAD-7 and WSAS means across time-points and by treatment group are presented in table 2 in the main paper.

Supplementary Table 1. PHQ-9, GAD-7 and WSAS linear mixed model fixed effect estimates at 8 weeks

Measure	Effect	*b*	SE *b*	95% CI
PHQ-9	Time	-2.42	0.52	-3.46, -1.39***
	Treatment Group	0.22	0.60	-0.95, 1.40
	Time x Treatment group	-2.75	0.64	-4.00, -1.50***
GAD-7	Time	-1.69	0.51	-2.69, -0.70*
	Treatment Group	0.12	0.54	-0.94, 1.18
	Time x Treatment group	-2.79	0.61	-4.00, -1.58***
WSAS	Time	-2.02	0.81	-3.62, -0.42*
	Treatment Group	-2.02	0.87	-3.73, -0.31*
	Time x Treatment group	-2.65	0.99	-4.59, -0.72*

Note: * p<.05, ** p<.001; *** p<.0001

Supplementary Table 2. Pooled PHQ-9, GAD-7 and WSAS marginal linear model fixed effect estimates at follow-up

Measure	Effect	*b*	SE *b*	95% CI
**PHQ-9**	Intercept	14.41	0.32	13.78, 15.03***
	Time – 8-weeks	-4.68	0.38	-5.41, -3.94***
	Time – 3-month	-5.65	0.40	-6.43, -4.87***
	Time – 6-month	-6.69	0.41	-7.49, -5.90***
	Time – 9-month	-7.33	0.43	-8.17, -6.50***
	Time – 12-month	-7.78	0.44	-8.65, -6.92***
**GAD-7**	Intercept	12.66	0.30	12.07, 13.25***
	Time – 8-weeks	-4.06	0.36	-4.77, -3.35***
	Time – 3-month	-4.72	0.38	-5.46, -3.97***
	Time – 6-month	-5.31	0.40	-6.10, -4.53***
	Time – 9-month	-5.95	0.40	-6.74, -5.16***
	Time – 12-month	-6.65	0.41	-7.45, -5.85***
**WSAS**	Intercept	17.35	0.46	16.45, 18.25***
	Time – 8-weeks	-4.24	0.56	-5.34, -3.14***
	Time – 3-month	-5.13	0.58	-6.27, -3.99***
	Time – 6-month	-6.44	0.61	-7.64, -5.23***
	Time – 9-month	-7.12	0.66	-8.41, -5.83***
	Time – 12-month	-7.48	0.66	-8.77, -6.19***

Note: *** p<.0001; Estimates pooled over 100 imputed datasets in line with Rubin's rules

2. M.I.N.I.7.0.2 outcomes

At 3-months follow-up, 179/241 treatment group participants (74%) completed the M.I.N.I.7.0.2 diagnostic interview. No statistically significant differences in baseline diagnoses were found between those who completed or failed to complete the interview (Supplementary Table 3). Changes in diagnostic status across the whole range of M.I.N.I.7.0.2 diagnoses from baseline to 3-months follow-up are presented in Supplementary Table 4.

Supplementary Table 3. Differences in baseline diagnoses between 3-month M.I.N.I.7.0.2 completers and non-completers

	3-month interview completed (n=179)	3-month interview not completed (n=62)	Statistic
M.I.N.I.7.0.2 diagnosis	143/179 (79.9%)	50/62 (80.6%)	$\chi2 = 0.17$; p = .99
MDD only	30/179 (16.8%)	7/62 (11.3%)	$\chi2 = 1.06$; p = .32
GAD only	29/179 (16.2%)	12/62 (19.4%)	$\chi2 = 0.32$; p = .69
SAD only	3/179 (1.7%)	1/62 (1.6%)	n/a
PD only	4/179 (2.2%)	2/62 (3.2%)	n/a
Comorbid anxiety disorder	12/179 (6.7%)	5/62 (8.1%)	$\chi2 = 0.13$; p = .77
Comorbid depressive & anxiety disorder	65/179 (36.3%)	23/62 (37.1%)	$\chi2 = 0.01$; p = .99

Note: n/a = test not applicable as expected cell counts too small

Supplementary Table 4. Change in M.I.N.I.7.0.2 diagnostic status from baseline to 3-months follow-up among interview completers (n=179)

		**3-months follow-up, n,%**							
		No diagnosis	MDD	GAD	SAD	PD	Comorbid anxiety disorders	Comorbid MDD & anxiety	**Total**
**Baseline, n,%**	No diagnosis	28/36 77.8%	3/36 8.3%	2/36 5.6%	0.0%	1/36 2.8%	0.0%	2/36 5.6%	36
	MDD	18/30 60.0%	2/30 6.7%	6/30 20.0%	0.0%	1/30 3.3%	0.0%	3/30 10.0%	30
	GAD	19/29 65.5%	3/29 10.3%	3/29 10.3%	2/29 6.9%	0.0%	2/29 6.9%	0.0%	29
	SAD	2/3 66.7%	0.0%	0.0%	1/3 33.3%	0.0%	0.0%	0.0%	3
	PD	2/4 50.0%	1/4 25.0%	0.0%	0.0%	1/4 25.0%	0.0%	0.0%	4
	Comorbid anxiety disorders	2/12 16.7%	0.0%	4 /12 33.3%	1/12 8.3%	3/12 25.0%	1/12 8.3%	1/12 8.3%	12
	Comorbid MDD & anxiety	30/65 46.2%	7/65 10.8%	8/65 12.3%	4/65 6.2%	2/65 3.1%	4/65 6.2%	10/65 15.4%	65
	**Total**	101	16	23	8	8	7	16	179

Note: MDD = Major Depressive Disorder; GAD = Generalized Anxiety Disorder; SAD=Social Anxiety Disorder; PD = Panic Disorder; Diagnostic categorization is mutually exclusive; individuals in the comorbid MDD & anxiety disorder category presented with one or multiple anxiety disorders

3. Cost-effectiveness analysis

3.1. EQ-5D-5L completion rates

Completion rates for the EQ-5D-5L are presented in Supplementary Table 5. Overall at baseline between intervention and control group, the EQ-5D-5L was completed by 238/241 (99%) and 117/120 (98%) participant,

respectively; at 8 weeks, this was 198/241 (82%) and 91/120 (76%) respectively; at final follow-up at 12 months in the intervention-arm, this was 172/241 (71%).

Supplementary Table 5. Mean and standard deviations for EQ-5D-5L cross-walk tariff scores by time-point in the complete case (CC) analysis

Measure	Time point, t_i	Intervention (1), N = 241				Control (0), N = 120				Mean Dif.
		Time-point (t_i)		Dif. time-points, $t_i - t_0$		Time-point (t_i)		Dif. time-points, $t_i - t_0$		
		N (%)	Mean (SD)	N (%)	Mean (SD)	N (%)	Mean (SD)	N (%)	Mean (SD)	1 – 0 (*p*)
EQ-5D-5L	t_0	238 (99)	0.656 (0.193)	-	-	117 (98)	0.645 (0.218)	-	-	0.011 (0.939)
cross-walk	t_1	198 (82)	0.723 (0.182)	196 (81)	0.065 (0.178)	91 (76)	0.676 (0.231)	89 (74)	0.020 (0.172)	0.048 (0.219)
tariff	t_2	186 (77)	0.753 (0.180)	184 (76)	0.090 (0.182)	-	-	-	-	-
	t_3	182 (76)	0.767 (0.212)	180 (75)	0.105 (0.217)	-	-	-	-	-
	t_4	176 (73)	0.779 (0.204)	174 (72)	0.112 (0.196)	-	-	-	-	-
	t_5	172 (71)	0.751 (0.201)	170 (71)	0.092 (0.215)	-	-	-	-	-

Labelling. t = time point, whereby: t_0 = baseline; t_1 = 8 weeks; t_2 = 3 months; t_3 = 6 months; t_4 = 9 months; t_5 = 12 months.
Footnote. N(%) states the number of people who completed the measure at the specific time-point i, or at two given time-points relative to t_0 (baseline), whereby i is any time-point denoted as 1 to 5.

3.2. EQ-5D-5L cross-walk tariff scores and subsequent QALYs by time-point and trial-arm

EQ-5D-5L cross-walk tariff scores in the complete case (CC) analysis are presented in Supplementary Table 5, with the cross-walk tariff score and subsequent QALY estimations for the intention-to-treat (ITT) analysis presented in Supplementary Table 6. The results in the CC and ITT analyses suggest a sustained health improvement over 12 months for those in the intervention-arm relative to baseline and a higher magnitude of health improvement over 8 weeks relative to the control group.

Supplementary Table 6. Mean and standard errors for EQ-5D-5L cross-walk tariff scores and resulting QALY values by time-point in the ITT and scenario analysis

Time-points	Intervention (1)		Control (0)		Dif. Means: (1) – (0)	
	Tariff, Mean (SE)	QALY, Mean (SE)	Tariff, Mean (SE)	QALY, Mean (SE)	Tariff, Mean (p-value)	QALY, Mean (p-value)
Baseline	0.6566 (0.0125)	-	0.6453 (0.0204)	-	0.0112 (0.6382)	-
8 weeks	0.7235 (0.0120)	0.1062 (0.0016)	0.6762 (0.0239)	0.1017 (0.0029)	0.0473 (0.0784)	0.0045 (0.1790)
3 months	0.7517 (0.0126)	0.1771 (0.0026)	*0.7224 (0.0165)*	*0.1689 (0.0047)*	*0.0294 (0.1354)*	*0.0082 (0.1279)*
6 months	0.7706 (0.0136)	0.3674 (0.0051)	*0.7430 (0.0156)*	*0.3521 (0.0086)*	*0.0275 (0.1670)*	*0.0153 (0.1226)*
9 months	0.7868 (0.0130)	0.5620 (0.0076)	*0.7656 (0.0131)*	*0.5406 (0.0120)*	*0.0213 (0.2224)*	*0.0214 (0.1288)*
12 months	0.7622 (0.0133)	0.7557 (0.0099)	*0.7465 (0.0108)*	*0.7297 (0.0148)*	*0.0157 (0.3147)*	*0.0260 (0.1378)*

Footnote. No data was collected for the control-group beyond 8 weeks; the values presented for the control-group and difference in means beyond 8 weeks are based on the scenario analyses and are presented here for descriptive purpose only to be cross-referenced with the QALY values presented in the scenario analyses.

3.3. Downstream care resource-use and associated costs by trial-arm

Within the intervention and control trial-arms, the modified client service receipt inventory (modified-CSRI) at 8 weeks was completed by 198/241 (82%) and 91/120 (76%) people and at baseline completed by 239/241 (99%) and 117/120 (98%) people, respectively. In the intervention-arm only at 6, 9, and 12 months, the modified-CSRI was completed by 182/241 (76%), 175/241 (72%), and 172/241 (71%) people, respectively. It is worth noting that resource-use at baseline describes resource-use 3-months pre-baseline (i.e. before intervention) and was used for the baseline adjusted cost analyses; resource-use over the 8-week treatment period is described by the modified-CSRI collected at 8-weeks. To give an indication of reported resource-use and associated costs over the 8 week treatment period (noting the CSRI asked about resource-use over the previous 3 months, and so these estimates include one month of pre-baseline data, which is a limitation of the analysis) and 3-months pre-baseline, these descriptive statistics (i.e. response rates, resource-use, and associated costs) by trial-arm in the CC analysis are presented in Supplementary Table 7 as reported at 8-week follow-up, and Supplementary Table 8 as reported at baseline. Mean costs and standard errors (SE) for the ITT analysis as reported at each time-point by trial-arm are presented in Supplementary Table 9; noting that no resource-use was reported for the control group beyond 8-week and so the costs presented in Supplementary Table 9 for the control-group are predicted based on the linear extrapolation described within the main manuscript. What is generally observed is a small cost difference between trial-arms as reported at 8-weeks (£3.16) which was also observed pre-baseline (£11.86) in the ITT analysis (Supplementary Table 9), and across resource-use parameters as reported in Supplementary Table 7 and Supplementary Table 8. This meant a small predicted difference in costs between trial-arms post-8-weeks which informed the scenario analyses (Supplementary Table 9).

Supplementary Table 7. Costs per CSRI resource-use parameter by trial-arm for the complete-case (CC) analysis collected at 8-weeks (3-month retrospective data)

Resource-use parameter	Intervention (N = 241)			Control (N= 120)			Dif. Means, Costs (£)
	All (N = 198), Mean (SE, median, min to max)	Resource-user N (%N)	Resource-user, Mean (SE, median, min to max)	All (N = 91), Mean (SE, median, min to max)	Resource-user N (%N)	Resource-user, Mean (SE, median, min to max)	
GP	44.78 (4.63, 31, 0 to 465)	120 (60.6%)	73.88 (6.36, 62, 31 to 465)	50.08 (4.72, 31, 0 to 186)	65 (50.1%)	70.11 (4.67, 62, 31 to 186)	-5.30
Practice Nurse	1.65 (0.31, 0, 0 to 19)	28 (14.1%)	11.64 (0.78, 9, 9 to 19)	1.43 (0.46, 0, 0 to 28)	11 (1.4%)	11.85 (1.82, 9, 9 to 28)	0.22
Physiotherapist	4.37 (1.80, 0, 0 to 289)	9 (4.5%)	96.18 (25.63, 96, 32 to 289)	2.47 (1.82, 0, 0 to 160)	3 (2.5%)	74.81 (42.75, 32, 32 to 160)	1.90
Specialist Nurse	1.38 (0.82, 0, 0 to 121)	3 (1.5%)	90.78 (17.47, 91, 61 to 121)	0.67 (0.47, 0, 0 to 30)	2 (0.7%)	30.26 (0.00, 30, 30 to 30)	0.71
Other doctor	2.17 (0.56, 0, 0 to 50)	16 (8.1%)	26.88 (2.56, 33, 17 to 50)	3.09 (0.89, 0, 0 to 33)	12 (3.1%)	23.43 (2.46, 17, 17 to 33)	-0.92
Podiatrist	0.34 (0.27, 0, 0 to 51)	2 (1.0%)	33.82 (16.91, 34, 17 to 51)	0.19 (0.19, 0, 0 to 17)	1 (0.2%)	16.91 (0.00, 17, 17 to 17)	0.15
Social worker	0.88 (0.62, 0, 0 to 87)	2 (1.0%)	87.30 (0.00, 87, 87 to 87)	1.92 (1.92, 0, 0 to 175)	1 (1.9%)	174.60 (0.00, 175, 175 to 175)	-1.04
Drug & Alcohol advisor	0.31 (0.31, 0, 0 to 61)	1 (0.5%)	60.52 (0.00, 61, 61 to 61)	0.00 (0.00, 0, 0 to 0)	0 (0.0%)	0.00 (0.00, 0, 0 to 0)	0.31
Other counsellor	4.20 (1.90, 0, 0 to 222)	6 (3.0%)	138.65 (31.21, 139, 55 to 222)	1.83 (1.36, 0, 0 to 111)	2 (1.8%)	83.19 (27.73, 83, 55 to 111)	2.37
Crisis team member	0.00 (0.00, 0, 0 to 0)	0 (0.0%)	0.00 (0.00, 0, 0 to 0)	0.00 (0.00, 0, 0 to 0)	0 (0.0%)	0.00 (0.00, 0, 0 to 0)	0.00
A&E	12.12 (3.23, 0, 0 to 320)	14 (7.1%)	171.43 (11.43, 160, 160 to 320)	17.58 (5.84, 0, 0 to 320)	9 (17.6%)	177.78 (17.78, 160, 160 to 320)	-5.46
Ambulance	2.97 (1.39, 0, 0 to 196)	5 (2.5%)	117.60 (19.60, 98, 98 to 196)	5.39 (2.81, 0, 0 to 196)	4 (5.4%)	122.50 (24.50, 98, 98 to 196)	-2.42
Inpatient/day-case	27.15 (13.99, 0, 0 to 1,769)	4 (2.0%)	1,343.75 (215.74, 1,432, 742 to 1,769)	34.25 (34.25, 0, 0 to 3,117)	1 (34.3%)	3,117.00 (0.00, 3,117, 3,117 to 3,117)	-7.10

Supplementary Table 8. Costs per CSRI resource-use parameter by trial-arm for the complete-case (CC) analysis collected at basline (3-month retrospective data)

Resource-use parameter	Intervention (N = 241), Costs (£)			Control (N= 120) , Costs (£)			Dif. Means, Costs (£)
	All (N = 239), Mean (SE, median, min to max)	Resource-user N (%N)	Resource-user, Mean (SE, median, min to max)	All (N = 117), Mean (SE, median, min to max)	Resource-user N (%N)	Resource-user, Mean (SE, median, min to max)	
GP	49.55 (3.09, 31, 0 to 465)	194 (81.2%)	61.04 (3.30, 62, 31 to 465)	50.87 (4.86, 31, 0 to 310)	91 (50.9%)	65.41 (5.34, 62, 31 to 310)	-1.32
Practice Nurse	2.57 (0.39, 0, 0 to 37)	47 (19.7%)	13.07 (0.97, 9, 9 to 37)	2.47 (0.65, 0, 0 to 37)	19 (2.5%)	15.19 (2.49, 9, 9 to 37)	0.10
Physiotherapist	2.95 (0.99, 0, 0 to 128)	12 (5.0%)	58.78 (11.05, 32, 32 to 128)	2.19 (0.93, 0, 0 to 64)	6 (2.2%)	42.75 (6.76, 32, 32 to 64)	0.76
Specialist Nurse	1.14 (0.63, 0, 0 to 121)	4 (1.7%)	68.09 (19.04, 61, 30 to 121)	0.78 (0.58, 0, 0 to 61)	2 (0.8%)	45.39 (15.13, 45, 30 to 61)	0.36
Other doctor	2.63 (0.67, 0, 0 to 99)	23 (9.6%)	27.33 (4.49, 17, 17 to 99)	2.55 (0.77, 0, 0 to 50)	12 (2.5%)	24.81 (3.22, 17, 17 to 50)	0.08
Podiatrist	0.07 (0.07, 0, 0 to 17)	1 (0.4%)	16.91 (0.00, 17, 17 to 17)	0.00 (0.00, 0, 0 to 0)	0 (0.0%)	0.00 (0.00, 0, 0 to 0)	0.07
Social worker	0.73 (0.45, 0, 0 to 87)	3 (1.3%)	58.20 (14.55, 44, 44 to 87)	1.87 (1.87, 0, 0 to 218)	1 (1.9%)	218.25 (0.00, 218, 218 to 218)	-1.14
Drug & Alcohol advisor	0.00 (0.00, 0, 0 to 0)	0 (0.0%)	0.00 (0.00, 0, 0 to 0)	0.52 (0.36, 0, 0 to 30)	2 (0.5%)	30.26 (0.00, 30, 30 to 30)	-0.52
Other counsellor	7.89 (3.02, 0, 0 to 555)	15 (6.3%)	125.71 (37.68, 55, 55 to 555)	4.74 (2.39, 0, 0 to 222)	5 (4.7%)	110.92 (30.38, 111, 55 to 222)	3.15
Crisis team member	1.10 (0.64, 0, 0 to 131)	4 (1.7%)	65.65 (22.74, 53, 26 to 131)	0.90 (0.71, 0, 0 to 79)	2 (0.9%)	52.52 (26.26, 53, 26 to 79)	0.20
A&E	14.73 (3.29, 0, 0 to 320)	20 (8.4%)	176.00 (11.01, 160, 160 to 320)	27.35 (16.74, 0, 0 to 1,920)	9 (27.4%)	355.56 (195.56, 160, 160 to 1,920)	-12.62
Ambulance	4.10 (1.40, 0, 0 to 196)	9 (3.8%)	108.89 (10.89, 98, 98 to 196)	4.19 (2.76, 0, 0 to 294)	3 (4.2%)	163.33 (65.33, 98, 98 to 294)	-0.09
Inpatient/day-case	35.88 (15.05, 0, 0 to 2,106)	7 (2.9%)	1,225.14 (252.68, 1,432, 0 to 2,106)	36.00 (25.67, 0, 0 to 2,443)	2 (36.0%)	2,106.00 (337.00, 2,106, 1,769 to 2,443)	-0.12

Supplementary Table 9. Mean and standard errors for downstream costs at all data collection periods by trial-arm for the intention-to-treat (ITT) and scenario analysis

Time-points	Mean (SE)		Mean (p-value)
	Intervention (1), N = 241	Control (0), N = 120	Dif. Means: (1) – (0)
Baseline	£122.91 (£18.24)	£134.77 (£33.67)	£-11.86 (0.7567)
8 weeks	£102.68 (£18.03)	£105.84 (£31.16)	£-3.16 (0.9295)
6 months	£118.65 (£21.20)	*£119.45 (£7.13)*	*£-0.80 (0.9686)*
9 months	£101.41 (£19.74)	*£103.08 (£10.57)*	*£-1.67 (0.9311)*
12 months	£92.14 (£15.84)	*£92.47 (£7.29)*	*£-0.34 (0.9812)*

Footnote. No data was collected for the control-group beyond 8 weeks; the values presented for the control-group and difference in means beyond 8 weeks are based on the scenario analyses and are presented here for descriptive purpose only to be cross-referenced with the QALY values presented in the scenario analysis for the CEA

3.4. Summary cost-effectiveness results by trial-arm and incremental results

Mean (bSE and 95% bCI) total costs and QALYs by trial-arm and difference in mean values between trial-arms for the CC, ITT, and scenario analyses are presented in Supplementary Table 10; incremental results which produced the incremental cost-effectiveness ratios (ICERs) and cost-effectiveness acceptability curves (CEACs; see Figure 4 in the main manuscript) are presented in Supplementary Table 11. As an example, in the intervention-group over 8-weeks in the CC (ITT) analysis: (1) for QALYs, mean unadjusted and adjusted total QALYs were 0.1063 and 0.1062 (0.1062 and 0.1057) compared to 0.1026 and 0.1027 (0.1017 and 0.1025) in the control-group, respectively, producing a mean QALY difference of 0.0037 or 0.0034 (0.045 or 0.0032); (2) for costs, mean unadjusted and adjusted total costs were £197.29 and £197.12 (£197.31 and £197.12) compared to £110.76 and £11.14 (£105.84 and £102.99) in the control-group, respectively, producing a mean cost difference of £86.53 or £85.67 (£91.47 and £95.72). Across all analyses, the iCBT intervention produced higher mean QALYs and total costs relative to the 8-week waiting list control; however, it is worth noting that as the intervention cost was £94.63, it is the intervention cost which is driving the main mean difference in total costs between trials-arms across all analyses with the intervention-group producing lower downstream care costs on average.

What is estimated across the different time-horizons for analyses (noting all data in the control-arm is predicted post-8-weeks, not observed) is that the ICER decreases as the time-horizon for analysis increases suggesting improved cost-effectiveness, which subsequently increases the probability of cost-effectiveness (see Supplementary Table 11). For example when accounting for baseline adjusted (BA) QALYs and BA costs in the ITT analysis, at 8-weeks the ICER is £29,764 with a probability of cost-effectiveness at £30,000 per QALY of 46.6%; however, in the ITT scenario analysis at 12 months, the ICER and probability of cost-effectiveness is £4,607 and 91.2%, respectively. Although the scenario analysis has its limitations, it is indicative of the potential cost-effectiveness of the iCBT intervention over 8-week waiting list control over the longer, 12-month time-horizon.

Supplementary Table 10. Unadjusted and adjusted cost and QALY estimates by trial-arm and difference in means between trial-arms

Analyses	Intervention group (1)					Control group (0)					Dif. Means (1) – (0) [a]			
	N (%)	Means	bSE*	95% bCI*		N (%)	Means	bSE*	95% bCI*		Means	bSE*	95% bCI*	
**Complete Case (CC) – 8 weeks**														
- Costs	194 (80)	£197.29	£18.09	£169.81	£246.64	88 (73)	£110.76	£35.63	£69.93	£262.30	£86.53	£40.03	-£50.35	£140.00
- BA Costs	194 (80)	£197.12	£17.53	£162.76	£231.48	88 (73)	£111.14	£23.72	£64.65	£157.64	£85.97	£29.12	£15.35	£131.25
- QALYs	194 (80)	0.1063	0.0018	0.1025	0.1096	88 (73)	0.1026	0.0034	0.0949	0.1084	0.0037	0.0038	-0.0034	0.0120
- BA QALYs	194 (80)	0.1062	0.0009	0.1045	0.1079	88 (73)	0.1027	0.0014	0.1000	0.1055	0.0034	0.0017	0.0003	0.0068
**Intention To Treat (ITT) – 8 weeks**														
- Costs	236 (98)	£197.31	£18.15	£166.11	£236.51	116 (97)	£105.84	£31.13	£62.99	£182.65	£91.47	£36.11	£9.62	£151.23
- BA costs	236 (98)	£198.71	£7.04	£188.78	£213.08	116 (97)	£102.99	£10.66	£94.17	£128.28	£95.72	£29.95	£30.06	£150.93
- QALY	236 (98)	0.1062	0.0017	0.1026	0.1092	116 (97)	0.1017	0.0027	0.0961	0.1067	0.0045	0.0031	-0.0013	0.0107
- BA QALYs	236 (98)	0.1057	0.0002	0.1053	0.1061	116 (97)	0.1025	0.0008	0.1014	0.1040	0.0032	0.0017	-0.0002	0.0066
**SCENARIO: Intention To Treat (ITT) – 6 months**														
- Costs	236 (98)	£315.96	£18.15	£166.11	£236.51	116 (97)	£225.29	£32.97	£178.67	£305.57	£90.67	£41.93	-£1.44	£165.44
- BA costs	236 (98)	£317.61	£9.44	£305.58	£339.72	116 (97)	£221.94	£12.31	£206.77	£258.77	£95.67	£35.79	£19.86	£164.21
- QALY	236 (98)	0.3674	0.0052	0.3565	0.3771	116 (97)	0.3521	0.0080	0.3356	0.3668	0.0153	0.0091	-0.0017	0.0337
- BA QALYs	236 (98)	0.3664	0.0014	0.3636	0.3690	116 (97)	0.3541	0.0032	0.3484	0.3600	0.0123	0.0070	-0.0016	0.0258
**SCENARIO: Intention To Treat (ITT) – 9 months**														
- Costs	236 (98)	£417.37	£38.87	£347.89	£496.87	116 (97)	£328.37	£39.02	£271.23	£421.04	£89.00	£53.97	-£23.69	£187.98
- BA costs	236 (98)	£419.61	£10.88	£404.99	£439.21	116 (97)	£323.82	£13.64	£304.40	£353.12	£95.79	£46.31	£3.67	£187.18
- QALY	236 (98)	0.5620	0.0078	0.5460	0.5771	116 (97)	0.5406	0.0112	0.5176	0.5613	0.0214	0.0130	-0.0029	0.0474
- BA QALYs	236 (98)	0.5607	0.0021	0.5569	0.5641	116 (97)	0.5434	0.0046	0.5356	0.5515	0.0172	0.0101	-0.0031	0.0368
**SCENARIO: Intention To Treat (ITT) – 12 months**														
- Costs	236 (98)	£509.51	£43.60	£431.09	£598.50	116 (97)	£420.85	£41.52	£357.35	£517.65	£88.66	£57.29	-£30.66	£192.46
- BA costs	236 (98)	£511.86	£14.60	£490.48	£534.47	116 (97)	£416.07	£16.91	£392.29	£449.65	£95.79	£49.41	-£2.60	£192.12
- QALY	236 (98)	0.7557	0.0101	0.7347	0.7753	116 (97)	0.7297	0.0137	0.7011	0.7550	0.0260	0.0163	-0.0049	0.0584
- BA QALYs	236 (98)	0.7540	0.0028	0.7485	0.7587	116 (97)	0.7332	0.0056	0.7233	0.7430	0.0208	0.0127	-0.0048	0.0454

Acronyms. BA: baseline adjusted; CE: cost-effectiveness; Dif. Mean: difference in mean values between trial arms; ICER: incremental cost-effectiveness ratio; Prob.: Probability; QALY: quality adjusted life year; bSE: Bootstrapped Standard Error. **Footnote.** The **scenario analyses** represents a scenario whereby the waiting list control participants health-related quality of life and care costs after 8 weeks up to twelve months followed the same trend as was observed in the intervention group; therefore, the difference in costs and QALYs are based on the observed values for the intervention group at each time-point but predicted values using regression analysis for the waiting-list control, the regression model for which is described in the methods section.

[a] Incremental results are the 'iCBT intervention' group (1; CC N = 194; ITT N = 236) minus the 'waiting-list control' group (0; CC N = 88; ITT N = 116)

Supplementary Table 11. Summary of incremental cost-effectiveness results for unadjusted and adjusted complete case, intention-to-treat and scenario analyses

Analyses: (1) – (0)[a]	Dif. Mean Costs (bSE)	Dif. Mean QALYs (bSE)	ICER	ICERs by CE plane quadrant (%)[b]						Prob. CE	
				SE (<£|>Q)	SW (<£|<Q)	NE (>£|>Q)	NW (>£|<Q)	S (<£)	E(>Q)	λ ≤£20k	λ ≤£30k
**Complete Case (CC) – 8 weeks**											
Cost / QALY	£86.53 (£40.03)	0.0037 (0.0038)	£23,385	2.4%	0.6%	81.3%	15.6%	3.0%	83.8%	44.1%	58.0%
BA cost / QALY	£85.97 (£29.12)	0.0037 (0.0038)	£23,233	0.1%	0.1%	83.7%	16.2%	0.1%	83.8%	42.1%	57.1%
Cost / BA QALY	£86.53 (£40.03)	0.0034 (0.0017)	£25,287	2.9%	0.1%	95.3%	1.7%	3.0%	98.2%	34.9%	58.6%
BA Cost / BA QALY	£85.97 (£29.12)	0.0034 (0.0017)	£25,123	0.1%	0.0%	98.1%	1.8%	0.1%	98.2%	31.0%	59.2%
**Intention To Treat (ITT) – 8 weeks**											
Cost / QALY	£91.47 (£36.11)	0.0045 (0.0031)	£20,310	1.7%	0.0%	91.5%	6.7%	1.8%	93.2%	47.9%	65.5%
BA cost / QALY	£95.72 (£29.95)	0.0045 (0.0031)	£21,253	0.5%	0.0%	92.7%	6.8%	0.5%	93.2%	45.4%	64.4%
Cost / BA QALY	£91.47 (£36.11)	0.0032 (0.0017)	£28,442	1.7%	0.1%	94.9%	3.3%	1.8%	96.6%	26.0%	49.8%
BA Cost / BA QALY	£95.72 (£29.95)	0.0032 (0.0017)	£29,764	0.5%	0.0%	96.0%	3.4%	0.5%	96.6%	21.0%	46.6%
**SCENARIO: Intention To Treat (ITT) – 6 months**											
Cost / QALY	£90.67 (£41.93)	0.0153 (0.0091)	£5,926	2.5%	0.1%	93.5%	4.0%	2.6%	96.0%	86.8%	90.6%
BA cost / QALY	£95.67 (£35.79)	0.0153 (0.0091)	£6,253	0.9%	0.0%	95.1%	4.0%	0.9%	96.0%	86.6%	90.6%
Cost / BA QALY	£90.67 (£41.93)	0.0123 (0.0070)	£7,374	2.5%	0.1%	93.4%	4.1%	2.6%	95.8%	85.0%	90.0%
BA Cost / BA QALY	£95.67 (£35.79)	0.0123 (0.0070)	£7,781	0.9%	0.0%	94.9%	4.2%	0.9%	95.8%	84.3%	89.6%
**SCENARIO: Intention To Treat (ITT) – 9 months**											
Cost / QALY	£89.00 (£53.97)	0.0214 (0.0130)	£4,159	5.7%	0.1%	89.7%	4.5%	5.8%	95.4%	89.3%	91.9%
BA cost / QALY	£95.79 (£46.31)	0.0214 (0.0130)	£4,476	2.2%	0.0%	93.2%	4.6%	2.2%	95.4%	89.2%	91.8%
Cost / BA QALY	£89.00 (£53.97)	0.0172 (0.0101)	£5,169	5.6%	0.2%	89.7%	4.5%	5.8%	95.3%	88.2%	91.3%
BA Cost / BA QALY	£95.79 (£46.31)	0.0172 (0.0101)	£5,564	2.2%	0.0%	93.1%	4.7%	2.2%	95.3%	87.6%	91.0%
**SCENARIO: Intention To Treat (ITT) – 12 months**											
Cost / QALY	£88.66 (£57.29)	0.0260 (0.0163)	£3,407	6.7%	0.2%	88.0%	5.1%	6.9%	94.7%	90.1%	92.0%
BA cost / QALY	£95.79 (£49.41)	0.0260 (0.0163)	£3,681	2.6%	0.1%	92.1%	5.2%	2.7%	94.7%	90.1%	92.0%
Cost / BA QALY	£88.66 (£57.29)	0.0208 (0.0127)	£4,264	6.6%	0.3%	88.1%	4.9%	6.9%	94.8%	89.0%	91.4%
BA Cost / BA QALY	£95.79 (£49.41)	0.0208 (0.0127)	£4,607	2.6%	0.1%	92.1%	5.2%	2.7%	94.8%	88.5%	91.2%

Acronyms. BA: baseline adjusted; CE: cost-effectiveness; Dif. Mean: difference in mean values between trial arms; ICER: incremental cost-effectiveness ratio; Prob.: Probability; QALY: quality adjusted life year; bSE: Bootstrapped Standard Error. **Footnote.** The **scenario analyses** represents a scenario whereby the waiting list control participants health-related quality of life and care costs after 8 weeks up to twelve months followed the same trend as was observed in the intervention group; therefore, the difference in costs and QALYs are based on the observed values for the intervention group at each time-point but predicted values using regression analysis for the waiting-list control, the regression model for which is described in the methods section.

3.5. Intervention cost

The unit costs and estimated cost for the iCBT intervention on aggregate and per person is presented within Supplementary Table 12. Overall, there were 256 trial participants who were assigned to use the iCBT intervention (see CONSORT diagram for patient flow during the trial time-horizon). The licence cost per person was £24 (Market based price for 2018/2019 NHS financial year for fixed volume of licences purchased). All initial assessments were conducted by Psychological Wellbeing Practitioner (PWPs) grade 5 and designed to take 40 minutes, with the majority of follow-up reviews being conducted also by PWPs grade 5 designed to take 15 minutes, and the majority of supervising staff being grade 6 for which 10 minutes were assumed to be proportioned to supervising staff specific to this trial and intervention. It should be noted that the number of follow-up reviews differed per person, and this is reflected within the aggregated and per person intervention cost (see Supplementary Table 12). The intervention cost in total consisted of: iCBT licence costs, £6,144 (£2400); initial patient assessment, £5,771 (£22.54); patient review, £10,435.34 (£40.76); supervision, £1,874 (£7.32). Overall, the intervention for the trial costed £24,224, which equates to a mean cost per person of £94.63.

Supplementary Table 12. Staff, resource-use, and unit costs used to calculate mean intervention cost per person

Parameter	Clients	Reviews	Unit cost[a] (per hour)	Time[b] (per hour)	Total cost	Per patient cost
iCBT licence costs	256	n/a	£24.00	n/a	£6,144.00	**£24.00**
**Initial assessment**						
PWP Grade 5	256	n/a	£33.81	0.67	£5,770.75	**£22.54**
**Reviews: PWP**						
Grade 4	7	18	£28.80	0.25	£129.58	
Grade 5	218	1054	£33.81	0.25	£8,909.73	
Grade 6	29	119	£43.63	0.25	£1,298.02	
Grade 7	1	5	£53.21	0.25	£66.51	
Grade 8a	1	2	£63.00	0.25	£31.50	
Sub-total: reviews	256	1198			£10,435.34	**£40.76**
**Supervision: senior IAPT staff**						
Grade 5	2	n/a	£33.81	0.167	£11.27	
Grade 6	246	n/a	£43.63	0.167	£1,788.87	
Grade 7	6	n/a	£53.21	0.167	£53.21	
Grade 8a	2	n/a	£63.00	0.167	£21.00	
Sub-total: supervision	256				£1,874.35	**£7.32**
**iCBT intervention cost**	256	n/a	n/a	n/a	£24,224.44	**£94.63**

[a] Staff costs based on hourly unit costs reported by Curtis and Burns(4).
[b] Time estimations for reviews are based on the suggested time spent during training procedures (see protocol), whereas initial assessment and supervision times are based on time spent in regular service procedures.

4. Further exploring the scenario analyses: extrapolation of outcomes from intervention to control-arm

4.1. Background

The base-case cost-effectiveness analysis in this study was focussed on a time horizon of 8-weeks, which represents the 8-week waiting period in the control-arm which reflects the 8-week treatment period for iCBT in the intervention-arm. Due in part to ethical reasons of withholding iCBT treatment in the control-arm beyond 8-weeks, the analysis which forms the comparative clinical and cost-effectiveness analysis could only be carried out over the 8-week period. However, for decision makers, it is useful to understand the potential for cost-effectiveness over a longer period of time e.g. one year.

In order to facilitate the discussion around the potential of this iCBT intervention to be cost-effective beyond 8-weeks, up to one year, scenario analyses were conducted. In the scenario analyses, outcomes observed beyond

8-weeks (at 3, 6, 9, and 12-months) in the intervention-arm were statistically applied to the control-arm's data for baseline and 8-weeks to extrapolate the control-arms outcomes beyond 8-weeks to the aforementioned time-points (i.e. 3, 6, 9, and 12-months).

4.2. Objectives

Within this section, we will further explain and explore:
(1) How the statistical extrapolation was applied using the observed data in the intervention and control-arms;
(2) The logical rationale behind the extrapolation;
(3) The results from the extrapolation in relation to the predicted outcomes and costs for the control-arm relative to intervention-arm and incremental differences;
(4) The strengths and limitations of the extrapolation;
(5) Aspects for decision makers to consider before deciding on if the scenario analyses can be considered to represent the potential of this iCBT intervention to be cost-effective beyond the 8-week period.

4.3. Methods: scenario analyses based on statistical extrapolation

The base-case cost-effectiveness analysis is conducted over the initial 8-weeks, as described in the main manuscript. Key aspects to reiterate include: (a) the cost-effectiveness analysis were conducted on a complete-case (CC) and intention-to-treat (ITT) basis; (b) baseline adjustments (BA) were made using ordinary least squares (OLS) linear regression models with covariates including trial-group and: (i) 3-months pre-baseline costs for costs; (ii) baseline EQ-5D-5L cross-walk score for QALYs; (c) non-parametric bootstrapping was used to calculate bootstrapped 95% confidence intervals (bCIs) and standard errors (bSE) around costs and effects, and for plotting cost-effectiveness acceptability curves (CEACs).

As part of the scenario analyses, regression-based extrapolations were used to estimate QALYs and costs beyond 8-weeks for the waiting-list control. That is, in the ITT dataset for the intervention-arm, OLS models were fitted to the (EQ-5D-5L) tariff score/(downstream) costs at 3 (not for costs), 6, 9, and 12 months as the response variable, independently, and tariff score/costs at baseline and 8 weeks as the explanatory variables; the predicted models were then fitted to the waiting-list control's data to estimate their potential tariff score/costs at the aforementioned time-points from which total costs and QALYs could be estimated, and CEA conducted.

To explain the scenario analyses step-by-step:

(1) for the **intervention-arm** ITT dataset (i.e. with missing cases already imputed), OLS models were fitted to the (EQ-5D-5L) tariff score/(downstream) costs at 3 (not for costs), 6, 9, and 12 months as the response variable, independently, and tariff score/costs at baseline and 8 weeks as the explanatory variables.
(2) the predicted models from the **intervention-arm** ITT dataset were then fitted to the **waiting-list control's** ITT dataset; that is, the predicted models were fitted to the tariff score/costs at baseline and 8 weeks as the explanatory variables for the waiting-list control data (as observed data were only available in the control-arm for baseline and 8-weeks; missing cases are already imputed at 8-weeks for the control-arm data before the models were fitted) and tariff score/costs were predicted at 3 (not for costs), 6, 9, and 12 months for the control-arm based on the mean linear patterns observed in the intervention-arm at these time points dependent on how the patient(s) tariff score/costs were at baseline and 8-weeks.
(3) After tariff score/costs were predicted at 3 (not for costs), 6, 9, and 12 months for the control-arm, patient-level total costs/QALYs were calculated across 3 (not for costs), 6, 9, and 12 months in the control-arm.
(4) With total costs and QALYs calculated at the new time-points in the control-arm, the CEA was conducted using the same methods as used in the base-case; that is, at each time-point:
    a. the CEA were conducted on a ITT basis (the CC analyses were dropped from the scenario analyses as ITT analyses are generally considered superior to CC analyses for informing decision makers when interpreting results from a trial);
    b. baseline adjustments (BA) were made using ordinary least squares (OLS) regression models with covariates including trial-group and: (i) 3-months pre-baseline costs for costs; (ii) baseline EQ-5D-5L cross-walk score for QALYs;
    c. non-parametric bootstrapping was used to calculate bootstrapped 95% confidence intervals (bCIs) and standard errors (bSE) around costs and effects, and for plotting cost-effectiveness acceptability curves (CEACs).

4.4. Rationale for the scenario analyses based on methods used

OLS models were chosen to simplify the assumptions behind the extrapolation such that the model and predicted outcomes could be readily understood by, and/or more easily explained to, decision makers wanting to interpret the scenario analyses.

The rationale behind the scenario analysis based on the linear model is as follows: by statistically applying the same mean linear trend as within the intervention-arm for the control-arm post 8-weeks, we can estimate the probability of cost-effectiveness under the scenario that the control-arm has the same linear trend as the intervention-arm post-8-weeks with the main differences being the patients' preference-based health status and external care costs as quantified at baseline and 8-weeks as observed within trial-arm at these time-points (i.e. the main between trial-arm comparative time-points of trial).

As we do not know what would have happened to the patients in the control-arm had they not received treatment post-8-weeks, but it seems reasonable to assume they may not have recovered as well as those in the treatment-arm in terms of their preference-based health status (on which the QALY is based) and could have required additional external healthcare (on which total costs is based) based on what is observed over the observed 8-week period or making assumptions based on evidence from the empirical literature, the linear extrapolation based on the observed trend in the intervention-arm seems like a conservative estimate for the extrapolation. If iCBT is then estimated to be cost-effective over the longer-term (i.e. beyond 8-weeks up to one year) under these conservative assumptions and predictions applied to the control-arm against which the intervention is judged to be cost-effective, then it seems logical to assume that the intervention could potentially be cost-effective at that ICER value/probability of cost-effectiveness or better had treatment been withheld for that time period rather than using the extrapolation.

By then applying methods such as the non-parametric bootstrap utilising the observed values in the intervention-arm and predicted post-8-week values in the control-arm, this allows us to take into account the uncertainty between-patients within-trial-arms to produce CEACs suggesting the probability of cost-effectiveness under these scenario analyses based on the linear extrapolation.

4.5. Results

Key results in terms of the probability of cost-effectiveness over 6, 9, and 12-months based on the extrapolation in the scenario analyses are described in the main manuscript. To reiterate:

"In the scenario analyses at 6, 9 and 12 months, the probability of cost-effectiveness increases as the time horizon increases (Figure 4). The probability of cost-effectiveness at 12-months ranges from 91.2% to 92.0% at £30,000, or 88.5% to 90.1% at £20,000, per QALY gained dependent on analysis conducted (Figure 4 only shows BA Costs / BA QALYs)."

Mean (bSE and 95% bCI) total costs and QALYs by trial-arm and difference in mean values between trial-arms for the CC, ITT, and scenario analyses are presented in Supplementary Table 10; incremental results which produced the incremental cost-effectiveness ratios (ICERs) and cost-effectiveness acceptability curves (CEACs; see Figure 4 in the main manuscript) are presented in Supplementary Table 11. Focussing specifically on comparing the BA ITT analyses at 8-weeks compared to the analyses post-8-weeks, it is worth noting that the ICER (= incremental mean costs / incremental mean QALYs) decreases as the time-horizon increases which is mainly attributable to the increasing incremental mean QALYs relative to relative stable mean cost differences (Table S17): 8-weeks, £29,764 (= £95.72/ 0.0032); 6-month, £7,781 (= £95.67 / 0.0123); 9-month, £5,564 (= £95.79 / 0.0172); 12-month, £4,607 (= £95.79 / 0.0208).

Although the observed BA cost differences at 8-weeks is similar to that predicted at 12-month (£95,72 Vs. £95,79) which is attributed mainly to the intervention cost (£94,63) as downstream external cost differences were predicted to be small at all time-points as was observed at baseline and 8-week (see Supplementary Table 9), the change in ICER comes down to the difference in BA QALY. For example, focussing on the BA QALYs at 8-weeks relative to 12-month (0.0032 Vs 0.0208), the BA QALY difference at 12-months is 6.5 times larger (i.e. 0.0208/0.0032 = 6.5) over a 6.5 times longer time period than that at 8-weeks (12 months is equivalent to 52 weeks such that: 52 weeks /8-weeks = 6.5).

It is worth noting, however, that between 8-weeks and 12-months the difference in tariff scores decrease between trial-arms (i.e. preference-based health status between trial-arms converge overtime) due to using a statistical extrapolation based on data in the intervention-arm for the control-arm, while the QALY differences based on the tariff scores at each time-point increases/diverge due to the area-under-the-curve (AUC; which uses data from the time-point of interest and all preceding time-points) method and time adjustment used to estimate the QALY. The difference in tariff scores between trial-arm at each time-point are shown in Supplementary Table 6; for example, the tariff score difference between trial-arms from 8-weeks to 12 months decreases from 0.0473 to 0.0157 while the QALY increases from 0.0045 to 0.0260 (note, in the previous paragraph describing

the ICER we focussed on the BA QALYs and BA costs; however, this examples focusses on the unadjusted estimates on which the BA is made such that the QALY and cost values differ in the examples provided, but the difference the BA makes to the unadjusted cost and QALY values are shown in Supplementary Table 10 and Supplementary Table 11). This change in tariff score and corresponding change in QALY is shown graphically in Supplementary Figure 2.

Focussing on Supplementary Figure 2: (a) Figure 2a shows an enhanced close-up of the change in tariff scores between time-points based on the observed and predicted values by narrowing the y-axis to focus on a specific tariff score range (i.e. 0.60 to 0.85); (b) Figure 2b shows the same change in tariff scores with the corresponding change in QALY values when the y-axis is set between 1 and 0, which for the tariff score, 1 represents a state equivalent to perfect health and 0 equivalent to dead as quantified at that time point, but for the QALY represents their time in that preference-based health state as reported at all time-points up to that specific time-point as based on the AUC method (i.e. at 12-months, this QALY represents being in a preference-based health state as reported at baseline, 8-week, 3, 6, 9, and 12-months). What can be observed in Figure 2a is that there is clear difference between tariff score trends between trial-arms from baseline to 8-weeks which is based on the observed and reported mean values within trial-arms at these time-points. After 8-weeks, the tariff score values are predicted for the control-arm based on the trend extrapolated from what was observed in the intervention-arm; as such, we see a much increased upward trend between 8-week and 3-months in the control-arm than between baseline and 8-week, and after 8-weeks the two trial-arms tariff scores follow the same overall trend while the difference in tariff scores between trial-arms narrows. The observed and predicted trend can also be seen in Figure 2b on a different y-axis scale, but with the corresponding QALY values by trial-arm also shown. As shown in Figure 2b, although the tariff score difference between trial-arms narrows as the time horizon increases suggesting the preference-based health status between trial groups converges overtime (which seems logical) the QALY difference increases which in turn will decrease the ICER value when total cost difference stay reasonably the same between trial-arms (as previously described). This changing difference in incremental costs relative to incremental QALYs as the time horizon of the analysis changes can also be observed on the cost-effectiveness planes (Supplementary Figure 3), which are presented based on the unadjusted (costs/QALYs) and BA (BA costs/BA QALYs) analyses with each dot on the scatter plot representing an ICER from the bootstrapped procedure used to produce the CEACs presented in Figure 1 of the main manuscript. Overall, this increasing incremental QALY with reasonably stable cost differences overtime decreases the ICER and increases the probability of cost-effectiveness as the time horizon of the analysis increases.

4.6. Discussion, strengths and limitations of the scenario analysis

The extrapolation used as part of the scenario analysis has its strengths and limitations which should be considered before interpreting the results of the analysis.

In regards to its strengths, the extrapolation can be considered a potentially conservative estimate of the QALY differences as previously described and, in this regard, can be used to make logical conclusions of the potential of iCBT to be cost-effective over the longer-term (i.e. post-8-week up to one year) based on the assumptions associated with the extrapolation on which the scenario analysis is based. For example, if decision makers think the difference in preference-based tariff scores between trial-group should be larger across time or at specific time-points based on professional opinion and/or other empirical evidence, then this would be reflected as increased QALY gains, which would decrease the ICER further and suggest a higher probability of cost-effectiveness than that presented as part of the scenario analyses. The exact amount by which the QALY would change is unknown; however, the results from this analysis can be used as a bench mark around which decision making can be informed around the potential of cost-effectiveness up to one year.

With no observed data beyond 8-weeks in the control-arm due to ethical reasons associated with the trial design, the main limitation is that the extrapolation is based on a simple linear model utilising the only observed data made available for the analysis on which trend lines beyond 8-weeks could be based: data from the intervention-arm. As such the trend lines are probably not wholly representative of the trajectory of the patients' health status and costs beyond 8-weeks in the control-arm and should be not considered as such, but instead (and as argued as a strength) these scenario analyses based on potentially conservative estimate can be used by decision makers to make an informed decision around the potential of the iCBT intervention to be cost-effective post-8-weeks up to 1 year against the waiting list control.

4.7. Summary of considerations for decision makers

The extrapolation is used as part of a scenario analysis and is not meant to fully represent the trajectory of the patient's health status and external costs of care (beyond the intervention cost) post-8-weeks in the control-arm. Instead, conservative estimates are used in order to estimate the potential of the iCBT intervention to be cost-effective beyond 8-weeks up to one year. Information of the estimated total costs, preference-based tariff scores

and associated QALYs, and resulting ICERs and CEACs are all presented within tables and/or figures within this appendix and main manuscript in order to make the analysis, extrapolation, and scenario analyses as transparent as possible.

If the decision maker is in agreement and believes that conservative estimates have been used to inform the scenario analyses and the analyses have been performed well (i.e. applying appropriate data and statistical methods as required for a suitable economic evaluation), then the results can be used as a benchmark of the potential of the iCBT intervention to be cost-effective beyond 8-weeks up to one year. However, if the decision-maker does not feel conservative estimates have been used and/or the analysis has not been conducted appropriately enough as to inform their decision making process, the scenario analysis should be disregarded and focus should be on the base-case cost-effectiveness analysis conducted up to 8-weeks at which time-point direct comparable observations could be made between trial-groups and a more traditional within-trial economic evaluation conducted.

5. Further treatment during follow-up period

Fifty five (55/241) intervention-arm participants (25.70%) either self-reported or were recorded by IAPT to have received further mental health treatment, as is presented in Supplementary Table 13. The table illustrates data for 60 participants, separated according to those who have one to three instances of further therapy across the follow-up period (3 months and onward). Of note, there is an overlap of 5 participants between CSRI and IAPTUS data, but this information cannot be reliably separated due to the self-report nature of the CSRI. Most participants reported 1 instance of therapy (n=42), although some participants had two (n=15) and few had up to three instances of therapy (n=3).

Supplementary Table 13. Description of further treatment across data available in the CSRI and IAPTus.

Data Source	Therapy Instance	Breakdown (instance)
CSRI (N=28) *[1]	3 reported instances of Therapy (n=2)	General Mental Health *[2] (n=6)
	2 Reported Instances of Therapy (n=7)	General Mental Health (n=7)
		Trauma Counselling (n=2)
		Not Specified (n=2)
		Therapy for Eating Disorder (n=1)
		Therapy for Body Dysmorphia (n=1)
		EAP (n=1)
	1 Reported Instance of Therapy (n=19)	General Mental Health (n=12)
		CBT (n=2)
		Relationship Counselling (n=1)
		Hypnotherapy (n=1)
		EAP (n=1)
		Integrated therapy for LTC (n=1)
		Bereavement Counselling (n=1)
IAPTus (n=32) *[3]	3 reported instances of Therapy (n=1)	Counselling (n=2)
		Bereavement Group (n=1)
	2 Reported Instances of Therapy (n=8)	CBT (n=12)
		Counselling (n=3)
		Guided Self-Help (N=1)
	1 Reported Instance of Therapy (n=23)	CBT (n=10)
		Counselling (n=6)
		Guided Self-Help (n=6)
		Integrated Therapy for LTC (n=1)

Note: CBT: Cognitive-Behavior Therapy; LTC: Long-term Condition; EAP: Employment Assistance Programme; *[1] The data taken from the CSRI is self-report from participants and is coded across several categories; *[2] The category 'General Mental Health' refers to unspecified therapy for depression, anxiety, stress and wellbeing; *[3] Five participants overlap between the self-reported CSRI data and electronic health record data.

Methods

1. Deviations from the original protocol submitted to the ethics committee

Research Ethics Committee amendments were requested and approved for each of the named changes below.

1. Change to schedule of several assessment scales and inclusion of new measures (AM01 06.06.2017).

I. EuroQoL Five Dimension Five Level version (EQ-5D-5L) was originally intended to be administered only at baseline and 8 weeks. It was included also at 3-, 6-, 9- and 12-month follow-up in order to assess cost-effectiveness during the follow-up period.

II. Client Service Receipt Inventory (CSRI) was originally scheduled to be administered at baseline, 8 weeks and at 6 months. It was included as well at 9- and 12-month follow-up to assess cost-effectiveness up to 12 months.

III. Emotion Regulation Questionnaire (ERQ) was added as a mechanism of change measure and administered at baseline and 8 weeks.

IV. Recovery in Quality of Life 10-item version (ReQoL-10) was added to aid validation of the scale and to conduct an additional economic analysis, and administered at baseline, 8 weeks, 3-, 6-, 9- and 12-month follow-up.

V. Positive Beliefs about Rumination scale (PBRS-A) was originally scheduled to be administered at baseline, 4 weeks and 8 weeks, but the 4 weeks assessment was later removed in order to reduce participant burden.

2. Amendment of payment method to participants (AM02 24.08.2017). Payment method was changed from cheques to One4All vouchers in order to have a more efficient payment to participants, avoiding issues around issuing and following cheques.

3. Amendment to the dropout questionnaire (AM03.15.06.2018). Originally it was described as only one question asking the reasons for dropout. In order to get a deeper understanding of the experience of participants who dropped out, an amendment was requested in order to introduce a short qualitative questionnaire at 6-month and 9-month follow-up. This expanded dropout questionnaire took around 15-20 minutes to complete and was conducted over the phone by a research team member.

4. Addition of a sub-study about implementation (AM04 08.11.2018). An amendment was requested in order to explore the experiences of PWPs associated with recruiting and supporting clients in the trial regarding the implementation of research-related procedures for this trial. Clinicians at the service were contacted via e-mail and if they were involved with the research procedures, they were requested to provide digital consent form and participate in an online administered questionnaire. The questionnaire took approximately 25 minutes to complete, and the research team reimbursed clinicians for their time with a £10 online shopping voucher.

Deviations from the original protocol not submitted to the ethics committee

5. In the original protocol submitted to the ethics committee, the sample size calculation was based on the use of F-tests to analyse the primary study outcomes (PHQ-9/GAD-7). However, following best practices we revised the statistical analysis plan and decided to apply Linear Mixed Models (LMM) instead of repeated-measures ANOVA for the primary outcomes analyses. This decision was based on LMM being more appropriate than ANOVA for different reasons:(1) (1) LMM are more robust to account for missing data, which is common in research designs with multiple timepoints, (2) ANOVA does not account for the correlation between repeated measurements obtained from the same individual whereas LMM is able to model this correlation through various covariance structures and thus account for it. The change in analytic strategy occurred during the write-up of the published protocol and, as such, happened prior to the unblinding of data. Nevertheless, this led to a discrepancy between the statistical methods informing the sample size calculations and those eventually applied, which could have compromised the power of the analyses we conducted. Post-hoc power calculations were conducted to ensure adequate power of primary analyses and address this limitation.

2. Deviations from the published protocol

6. A recruitment period of 9 months to reach the target of 360 participants was estimated. However, the recruitment period was extended one month in order to achieve the sample size.

7. Monetary incentives offered to participants in the trial were missed to be reported in the published protocol. Specifically, the control group participants received £60 for completing measures at 8-weeks from baseline. Experimental group participants received up to £60 at 3-month follow-up and up to £37.50 at 12-month follow-up depending on how many assessments they had completed in the months previously (£10 paid per completed

assessment across six assessments and accumulatively paid out at 3-month follow-up; £12.50 paid per completed assessment across three assessments and accumulatively paid out at 12-month follow-up).

8. ‘Caseness’ is established by scoring PHQ-9 ≥ 10 and/or GAD7 ≥ 8, and therefore, recovery is determined by patients moving from ‘caseness’ at the beginning of the intervention to ‘non-caseness’ at the end of treatment. In the original published protocol, the threshold for ‘caseness’ was wrongly stated at ≥9 on the PHQ-9 and ≥8 on the GAD-7.

3. Declaration of reported measures (see protocol for a detailed description of the measures)

I. The present paper answers the two main research questions of the trial protocol, namely, effectiveness and cost-effectiveness of the intervention up to 12 months follow-up by using the standards provided by the National Institute for Health and Care Excellence (NICE) guidelines. Hence, effectiveness is accounted by the principal scales used within NHS IAPT that measure depression (PHQ-9), anxiety (GAD-7) and functional impairment (WSAS), which are part of the Minimum Dataset[1]. Cost-effectiveness is measured using the EQ-5D-5L (preference-based generic health status) crosswalk which is part of NICE’s current reference case for economic evaluations(2,3), and a modified Client Service Receipt Inventory (CSRI), which records self-reported care resource-use over the previous 3-months that is routinely collected within IAPT.

II. Recovering Quality of Life – 10-item version (ReQoL-10). The preference-based scoring algorithm for this measure has not yet been finalised nor published, the psychometric validity for which still needs to be tested. Hence, the authors are unable to report on it here, but rather base the cost-effectiveness results on the EQ-5D-5L, which is currently part of the NICE reference case for economic evaluations. Psychometric validity of the ReQoL-10 and EQ-5D-5L against the GAD-7 and PHQ-9 will be assessed in a separate paper, publication of which will be under embargo until the preference-based algorithm for the ReQoL-10 is finalised and published.

III. Diagnostic-specific measures (PSWQ, PDSS, SPIN and HAI) were collected because they are available for optional administration by PWPs within IAPT. However, these questionnaires were administered inconsistently throughout the trial, rendering them unusable for the trial. For example, in many occasions these questionnaires were administered at 8 weeks without being administered at baseline. For this reason, these questionnaires will not be analysed nor reported in this or other studies.

IV. IAPT Phobia scale is administered within the Minimum Dataset of IAPT but it is not an empirically validated measure of phobic anxiety. Within IAPT, the IAPT Phobia scale has the purpose to identify individuals that fall below the clinical threshold on the PHQ-9 and GAD-7 but present with significant phobic anxiety – individuals that were excluded from the current trial according to inclusion criteria anyway. Thus, the IAPT Phobia Scale was deemed irrelevant in the context of this trial and it will only be used to assess the psychometric validity of the ReQoL-10, which is reported in point II.

V. Measures related to the exploration of mechanisms of change/maintenance of effects (STAR-C, STAR-P, ERQ, PBRS-A, FATS, therapist behaviors, expectancy/credibility questionnaire, CBT skills usage) will be analysed and reported elsewhere given that they have been included at a more exploratory level and respond to different research objectives than those addressed within this study.

VI. The dropout questionnaire only included one question about the reasons for dropout from treatment. However, an ethics amendment was requested after the trial had started to conduct qualitative interviews with some of these participants in order to delve deeper into the experience of participants who dropped out. Due to the depth of the interviews and the qualitative methodology utilised, it was decided to report their outcomes elsewhere to allow for a higher level of detail then.

4. Cost-effectiveness analysis: estimating intervention and downstream resource-use and costs

Estimating the iCBT intervention cost

Intervention resource-use is based on: (1) iCBT platform licence cost; (2) initial patient assessment to use the platform by a Psychological Wellbeing Practitioner (PWP pay-grade 5); (3) patient review (PWP pay-grades 4 to 8a); (4) supervision of the PWPs (senior staff [i.e. senior PWPs, counsellors, clinical psychologists] pay-grade 5 to 8a). The staff involved in each of the tasks is based on trial records. Time assumptions for completing each task is based on the average length of time spent for each task for clinical staff during the trial. The time for supervision and initial assessment is based on service procedures executed during the trial period. Care professional staff costs for 2017/18 were obtained from Curtis and Burns(4) (a commonly used source of unit

costs for health and social care within the UK) and applied per patient for the intervention, which was subsequently aggregated and with the mean cost per patient representing the intervention cost used in the cost-effectiveness analysis. All unit costs associated with the iCBT intervention are presented alongside the intervention cost calculation within the eResults.

Estimating downstream resource-use and associated costs

Self-reported resource-use was collected using an adapted Client Service Receipt Inventory (adapted-CSRI), which is routinely collected within IAPT services (the adapted-CSRI is presented in Supplementary

Supplementary Figures

Supplementary Figure 1). It should be noted that there are a range of (adapted) 'CSRIs' available for use, accessible via the Database of Instruments for Resource Use Measurement (DIRUM) website: www.dirum.org. The use of the term 'adapted' is to distinguish the use of a CSRI from the original developed by Beecham & Knapp in 1992;(5) since then, the CSRI has been adapted many times with 200 versions believed to have been used, the use of which is described and discussed by Franklin & Thorn.(6) This adapted-CSRI was collected at two time-points in both trial-arms: baseline and 8 weeks; and an additional three time-points in the intervention-arm: 6 months, 9 months, and 12 months. At all time-points the adapted -CSRI requests participants to report their care resource-use over the previous 3 months. At 8 weeks, the adapted-CSRI should have asked participants about their resource-use over the previous 8 weeks (i.e. from baseline to 8 weeks) in both trial-arms and at 6-month the adapted-CSRI should have asked about resource-use over the previous 4 months (i.e. 8 weeks up until 6 months). These were trial-oversights which means that resource-use between 8 weeks and 3 months is not reported, the implications of which for the CEA is discussed as a limitation. Unit costs were attached per unit of resource-use, as presented and referenced within Supplementary Table 14 and Supplementary Table 15.

Supplementary Table 14. Unit costs for care professionals

CSRI description	Assumed location	Unit cost description	Unit cost	Unit cost time metric	Referenced/ assumed time	Applied cost	Source
General Practitioner (GP)	GP Practice	GP (including direct care cost) within GP surgery	£31.00	9.22 minutes	9.22	£31.00	Cost(4) Time(4)
Practice nurse	GP Practice	Nurse (Practice)	£36.02	60 minutes	15.5	£9.31	Cost(4) Time(11)
Physiotherapist	Hospital	Physiotherapist (band 5)	£34.60	60 minutes	55.6	£32.06	Cost(4) Time(12)
Specialist Nurse (e.g. cardiac nurse, diabetes nurse)	Hospital	Nurse specialist (band 6)	£45.40	60 minutes	40	£30.26	Cost(4) Time(13)
Non-GP doctor for physical health (e.g. cardiologist, gastroenterologist, oncologist)	Hospital	Consultant medical	£107.65	60 minutes	9.22 (≈GP)	£16.54	Cost(4) Time(4)
Podiatrist	Community	Podiatrist (band 5)	£33.81	60 minutes	30	£16.91	Cost(4) Time(14)
Social Worker	Community	Social worker (adult services)	£43.65	60 minutes	60	£43.65	Cost(4) Time(13)
Drug & alcohol advisor	Community	Alcohol health worker/ Alcohol liaison nurse/ Substance misuse nurse	£45.40	60 minutes	40 (≈Specialist Nurse)	£30.26	Cost(4) Time: N/A
Other counsellor / therapist / clinical psychologist (Outside the IAPT Service)	Hospital	Psychologist (band 7)	£55.46	60 minutes	60	£55.46	Cost(4) Time(15)
Home treatment / Crisis team member/ Assertive outreach team member/ Community mental health team member e.g. Psychiatrist, Mental health nurse (CPN)	Community	Community mental health team for adults with mental health problems (cost per team member; weighted 31% community nurse)	£39.39	60 minutes	40 (≈Specialist Nurse)	£26.26	Cost(16) Time(13)

Acronyms. CPN, Community Psychiatric Nurse; GP, General Practitioner.

[a] For these care professionals a band wage was assumed and applied based on 2017/18 unit costs obtained from the Excel database compiled by Curtis and Burns(4), sheet titled "Unit costs - professionals".

[b] The unit cost for 2016/17 was obtained for Curtis and Burns(16) from and then inflated to 2017/18 using the NHS staff pay index as presented within Curtis and Burns(4)

Supplementary Table 15. Unit costs for hospital and ambulance resource-use

CSRI description	Assumed location	Unit cost description	Unit cost	Unit cost metric	Applied cost	Source
A&E	Hospital	Accident & Emergency	£160	Per visit	£160	(17)
Inpatient (average)	Hospital	Non-elective inpatient (average LOS: 6 days)	£3,117	6 days	LOS dependent	(17)
- Excess bed days	Hospital	Non-elective inpatient (excess bed days)	£337	1 day (extra)	LOS dependent	(17)
- Day cases	Hospital	Day case	£742	1 day (0 nights)	£742	(17)
Ambulance	Community	Ambulance	£98	Per trip	£98	(17)

Acronyms. A&E, Accident & Emergency; LOS, Length of Stay.

Inpatient stay cost calculation: The average length of stay (LOS) across all recorded inpatient stays within NHS Improvement(17) "2017/18 reference cost data" is 6 days. An excess bed day cost is added/subtracted equivalent to the number of inpatient days reported which are more/less than 6 days e.g. if a LOS was reported to be 8 days, this was estimated to cost £3791 = £3,117 + (2 days * £337); if LOS was reported to be 3 days, this was estimated to cost £2,106 = £3,117 - (3 days * £337).

5. Missing data analysis

In terms of demographic variables, missing data was under 3% (12/361) across all variables, whereas for outcomes variables (PHQ-9, GAD-7, WSAS) it ranged between 19.9% (289/361) at 8-weeks (across both groups) and 28.2% (173/241) at 12-month follow-up (in treatment group only as control not followed-up). Little's 'Missing Completely At Random' (MCAR) test confirmed that at 8-weeks and across both groups data was missing completely at random ($\chi2(4)=1.66$, p=.80). In analysing missing data across the follow-up period and including all data from demographic and outcome variables from the treatment group only, Little's MCAR test suggested that data may not be missing completely at random ($\chi2(291)=327.92$, p=.067).

Further exploring these findings, Chi-squared tests, independent t-tests and Mann-Whitney tests were used to assess differences between participants who completed or failed to complete outcome measures. At 8-weeks and in line with Little's test, no statistically significant differences between those who did or did not complete measures were detected (see Supplementary Table 16). Given this strong evidence that data was missing completely at random at 8-weeks, linear mixed models (LMMs) were deemed suitable in analysing the primary study outcomes. Given potential issues around misspecification in multiple imputation and the robustness of LMMs to missing data (7), it was decided to proceed without the imputation of missing data in this instance.

Supplementary Table 16. Differences between 8-week responders and non-responders

		No. (%)		Tests statistic & p-value
		**Completed 8-week measures (N=289)**	**8-week measures not completed (N=72)**	
Treatment group	Treatment	198/241 (85.3%)	43/241 (14.6%)	$\chi2(1)=2.01.$; p=.16
	Waiting	91/120 (75.8%)	29/120 (24.2%)	
Age	Median (IRQ)	29 (18)	28 (19)	U=9862.0; p=.49
Gender	Female	210/258 (81.4%)	48/258 (18.6%)	$\chi2(1)=1.02$; p=.31
	Male	79/103 (76.7%)	24/103 (23.3%)	
Ethnicity	White	264/304 (80.9%)	58/304 (19.1%)	$\chi2(1)=1.28$; p=.26
	Non-white or mixed	37/50 (74%)	13/50 (26%)	
Employment status	In employment	213/269 (79.2%)	56/269 (20.8%)	$\chi2(1)=0.50$; p=.55
	Not in employment	76/92 (82.6%)	16/92 (17.4%)	
Psychoactive medication	Taking	129/158 (81.6%)	29/158 (18.3%)	$\chi2(1)=0.33$; p=.59
	Not taking	160/202 (79.2%)	42/202 (20.8%)	
Baseline MINI	Diagnosis given	228/290 (78.6%)	62/290 (21.4%)	$\chi2(1)=1.90$; p=.19
	No diagnosis	61/71 (85.9%)	10/71 (14.1%)	
Baseline PHQ-9	Mean, SD	14.29 (5.10)	14.49 (4.56)	t(359)= -0.98; p=.77
Baseline GAD-7	Mean, SD	12.61 (4.54)	12.68 (4.45)	t(359)= -0.54; p=.90
Baseline WSAS	Mean, SD	17.81 (7.42)	18.91 (7.42)	t(359)= -1.13; p=.26

Regarding follow-up, the most poignant contrast between the two most prevalent missingness patterns of participants either completing all follow-up measures (n=149) or completing none (n=35) was selected (see Supplementary Table 17). This revealed that significantly more females than males completed all follow-up measures ($\chi2(1)=5.37$, p=.03). There also appeared to be an effect of ethnicity on participants follow-up completion rate; however, this remained an insignificant trend ($\chi2(1)=3.79$, p=.08). Thus, there seemed to be some evidence that the trend towards significance in Little's test may be explained by these two variables. Given the possibility that data was not missing completely at random at follow-up and marginal model requirements for MCAR missing data, it was decided to implement multiple imputation via multilevel joint modelling(8) in this

instance. The R package 'mitml'(9) was used to impute data prior to fitting marginal models to the follow-up data through the 'geepack' package(10) and pooling effect estimates according to Rubin's rules.

Supplementary Table 17. Differences between those who completed all follow-up measures and those who completed none

		No. (%)		Tests statistic & p-value
		**Completed all follow-up measures (N=149)**	**Completed none of the follow-up measures (N=35)**	
Age	Median (IRQ)	29 (18)	28 (19)	U=2444.5; p=.56
Gender	Female	114/134 (85.1%)	20/134 (14.9%)	$\chi 2(1)=5.37$; p=.03
	Male	35/50 (70%)	15/50 (30%)	
Ethnicity	White	125/158 (79.1%)	33158 (20.9%)	$\chi 2(1)=3.79$; p=.08
	Non-white or mixed	21/22 (95.4%)	1/22 (4.5%)	
Employment status	In employment	113/143 (79%)	30/143 (21%)	$\chi 2(1)=1.60$; p=.26
	Not in employment	36/41 (87.8%)	5/41 (12.2%)	
Baseline psychoactive medication	Taking	62/74 (83.8%)	12/74 (16.2%)	$\chi 2(1)=0.63$; p=.43
	Not taking	87/110 (79.1%)	23/110 (20.9%)	
Baseline MINI	Diagnosis given	118/147 (80.2%)	29/147 (19.7%)	$\chi 2(1)=0.24$; p=.65
	No diagnosis	31/37 (83.8%)	6/37 (16.2%)	
Baseline PHQ-9	Mean, SD	14.21 (4.86)	14.26 (5.30)	t(182)=-0.05; p=.96
Baseline GAD-7	Mean, SD	12.46 (4.74)	13.37 (4.51)	t(182)=-1.02 p=.30
Baseline WSAS	Mean, SD	17.41 (7.34)	16.31 (6.64)	t(182)=0.81 p=.42

Supplementary Figures

Supplementary Figure 1. Modified Client Service Receipt Inventory (CSRI) used within Berkshire IAPT service

Are you completing this questionnaire *(please select one):*

- □ **Before** you start your treatment with IAPT
- □ **Immediately after** have you have completed your treatment with IAPT
- □ **At Follow-Up** (i.e. some time after completing your treatment)

1. In the last 3 months, what face-to-face appointments have you had with health professionals outside of the IAPT therapy service? (*Note: only record one-to-one contacts here; see next questions for A&E)*

**Care provider**	**Have you seen any of the following healthcare professionals in the last three months ?**<br>(circle)	**Usual location (Please write down one of the following numbers)**<br>1 = GP practice<br>2 = Community centre<br>3 = Hospital utpatient<br>4 = Own home	**No. of contacts in last 3 months**	**Reason for attending**
General practitioner (GP)	*No / Yes*			
Practice Nurse	*No / Yes*			
Physiotherapist	*No / Yes*			
Specialist Nurse (e.g. cardiac nurse, diabetes nurse)	*No / Yes*			
Doctor other than GP for a physical health problem (e.g. cardiologist, gastroenterologist, oncologist etc)	*No / Yes*			
Podiatrist	*No / Yes*			
Social Worker	*No / Yes*			
Drug & alcohol advisor	*No / Yes*			
Other counsellor / therapist / clinical psychologist (Outside the IAPT Service)	*No / Yes*			
Home treatment / Crisis team member/ Assertive outreach team member/ Community mental	*No / Yes*			

health team member e.g. Psychiatrist, Mental health nurse (CPN)				

2. In the last 3 months, **how many times have you attended A & E** (Accident & Emergency)?

………………………………………….

3. In the last 3 months, **have you been admitted to hospital as an inpatient**? *Yes* **or** *No*

(please circle)

If yes:

Name of hospital	***Reason for admission***	***How many days were you in hospital for you?***	***Do you recall the admission date?***

4. Have you needed to call an ambulance in the last 3 months? *Yes* **or** *No (please circle)*

If yes:

**How many times have you needed an ambulance in the last 3 months?**	**Reason for calling the ambulance**

Supplementary Figure 2: Observed and predicted EQ-5D-5L tariff scores and subsequent QALY values by trial-arm as plotted at baseline (0 months), 8-weeks, 3, 6, 9, and 12 months.

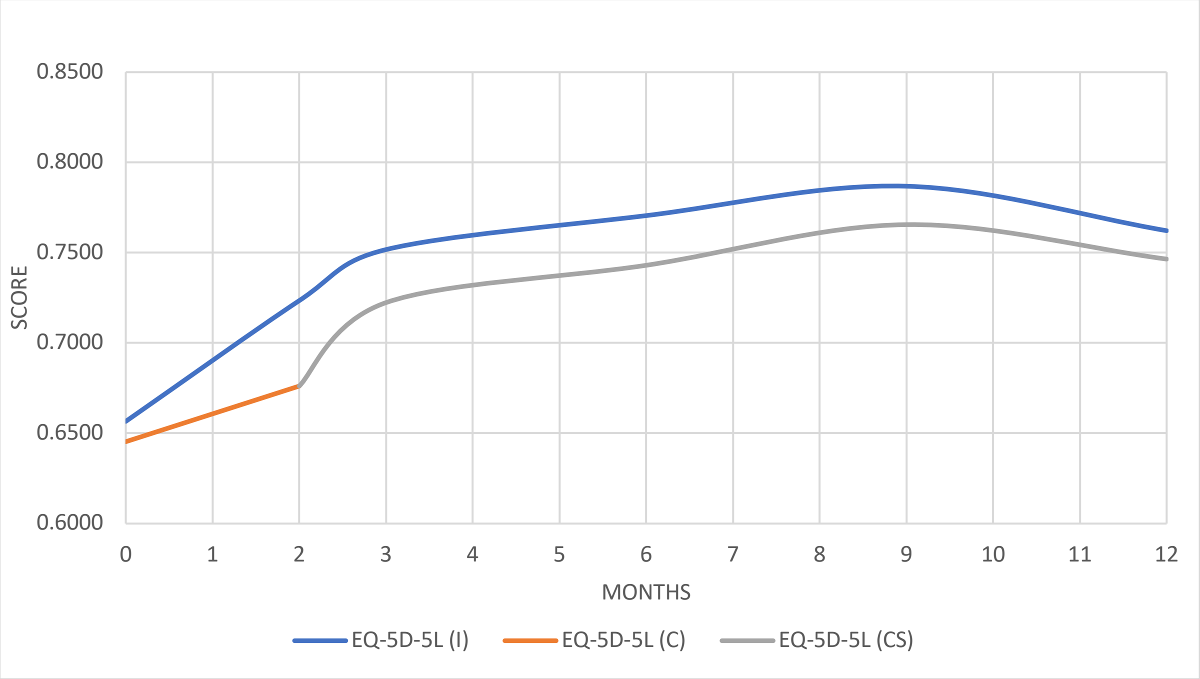

Supplementary Figure 2a: EQ-5D-5L tariff scores observed in the intervention (I) and control (C) trial-arms, and predicted in the control-arm beyond 8-weeks for the scenario analyses (CS). Note, the y-axis has been restricted to between 0.6 and 0.85 to better present the trend change in tariff scores between time-points and between trial-arms.

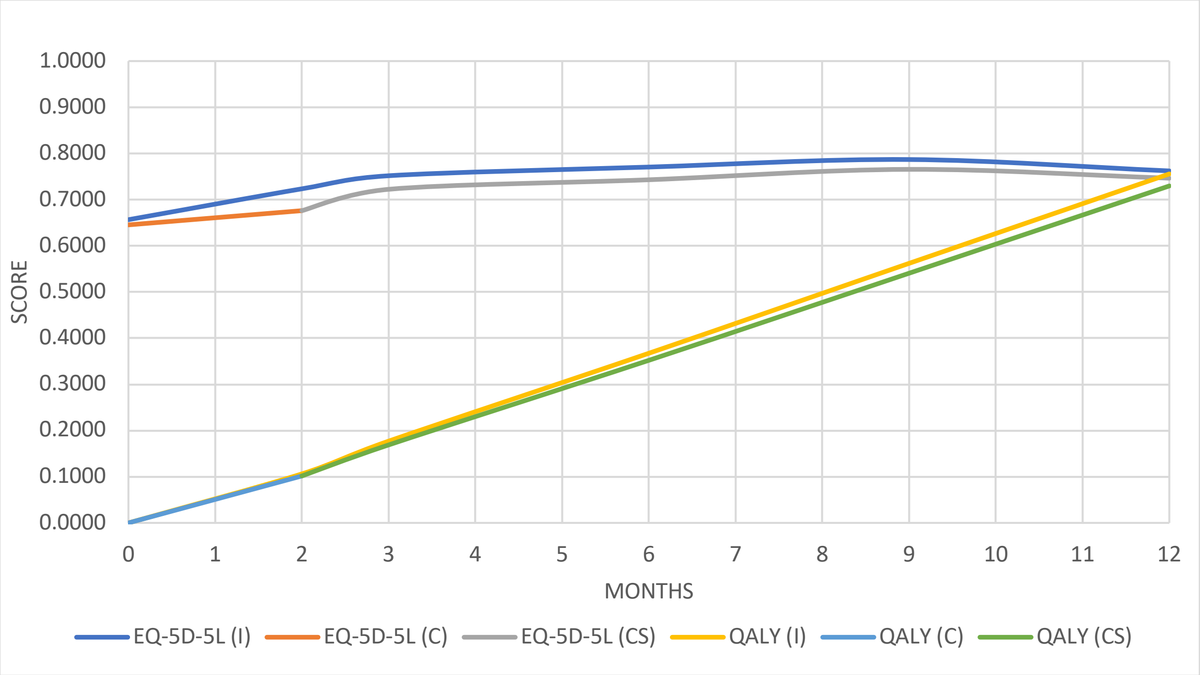

Supplementary Figure 2b: EQ-5D-5L tariff and subsequent QALY scores observed in the intervention (I) and control (C) trial-arms, and predicted in the control-arm beyond 8-weeks for the scenario analyses (CS).

Supplementary Figure 3: cost-effectiveness planes showing difference in unadjusted and baseline adjusted (BA) QALYs (x-axis) and costs (y-axis) between trial-arms across 8-weeks, 6, 9, and 12-months

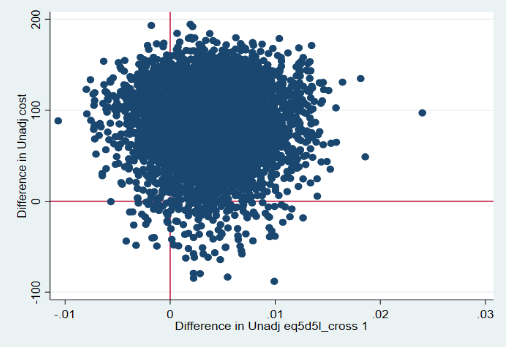

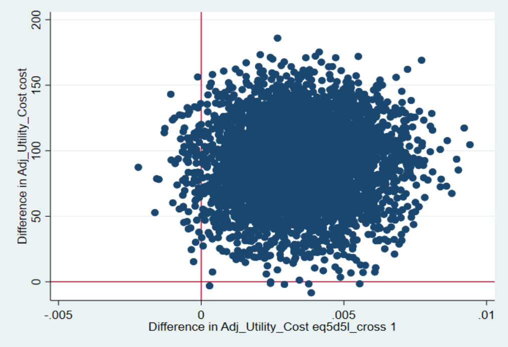

(a) 8-weeks: unadjusted QALYs & costs *(b) 8-weeks: BA QALYs & BA costs*

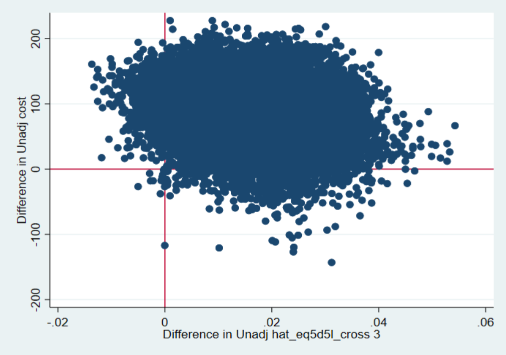

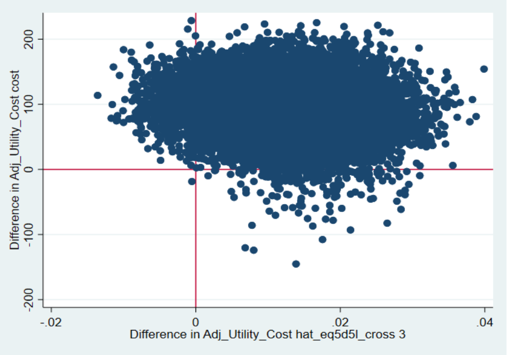

(c) 6-month: unadjusted QALYs & costs *(d) 6-month: BA QALYs & BA costs*

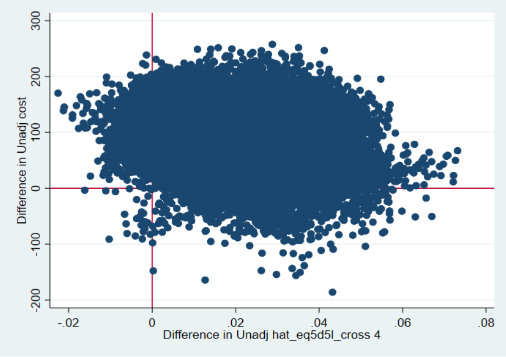

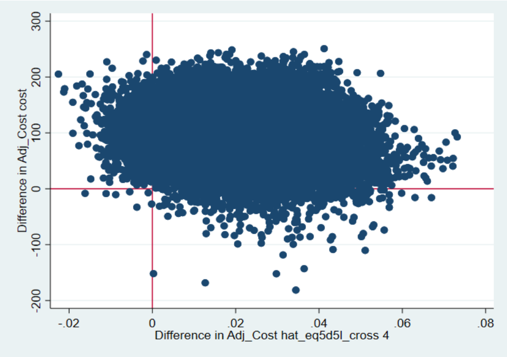

(e) 9-month: unadjusted QALYs & costs *(f) 9-month: BA QALYs & BA costs*

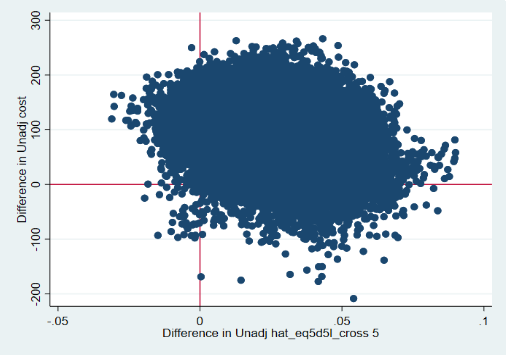

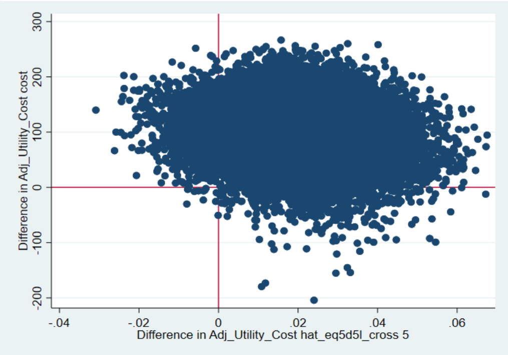

(g) 12-month: unadjusted QALYs & costs *(h) 12-month: BA QALYs & BA costs*

References

1. West BT, Welch KB, Gałecki AT, Gillespie BW. Linear mixed models : a practical guide using statistical software. Boca Raton, Florida: CRC press; 2014. 440 p.

2. National Institute for Health and Care Excellence. Position statement on use of the EQ-5D-5L valuation set for England (updated November 2018) [Internet]. NICE technology appraisal guidance. NICE; 2018. Available from: https://www.nice.org.uk/about/what-we-do/our-programmes/nice-guidance/technology-appraisal-guidance/eq-5d-5l

3. National Institute for Health and Care Excellence. Guide to the methods of technology appraisal [Internet]. Process and methods. London, UK; 2013. Available from: https://www.nice.org.uk/process/pmg9/resources/guide-to-the-methods-of-technology-appraisal-2013-pdf-2007975843781

4. Curtis L, Burns A. Unit Costs of Health and Social Care 2018. Project Report [Internet]. Canterbury: University of Kent; 2018. Available from: https://kar.kent.ac.uk/70995/

5. Beecham J, Knapp M. Costing psychiatric interventions. In: Thornicroft G, Brewin C, Wing J, editors. Measuring mental health needs. London: Gaskell; 1992.

6. Franklin M, Thorn J. Self-reported and routinely collected electronic healthcare resource-use data for trial-based economic evaluations: the current state of play in England and considerations for the future. BMC Med Res Methodol [Internet]. 2019 Dec 9 [cited 2020 Jan 31];19(1):8. Available from: https://bmcmedresmethodol.biomedcentral.com/articles/10.1186/s12874-018-0649-9

7. Sullivan TR, White IR, Salter AB, Ryan P, Lee KJ. Should multiple imputation be the method of choice for handling missing data in randomized trials? Stat Methods Med Res. 2018 Sep;27(9):2610–26.

8. Huque MH, Carlin JB, Simpson JA, Lee KJ. A comparison of multiple imputation methods for missing data in longitudinal studies. BMC Med Res Methodol [Internet]. 2018 Dec 12 [cited 2020 Jan 30];18(1):168. Available from: https://bmcmedresmethodol.biomedcentral.com/articles/10.1186/s12874-018-0615-6

9. Grund S, Lüdtke O, Robitzsch A. Multiple Imputation of Multilevel Missing Data. SAGE Open [Internet]. 2016 Oct [cited 2020 Jan 30];6(4):215824401666822. Available from: http://journals.sagepub.com/doi/10.1177/2158244016668220

10. Halekoh U, Højsgaard S, Yan J. The R package geepack for generalized estimating equations. J Stat Softw. 2006;15(2):1–11.

11. Curtis L, Burns A. Unit Costs of Health and Social Care 2015 [Internet]. University of Kent, Canterbury: Personal Social Services Research Unit (PSSRU); 2015. Available from: https://www.pssru.ac.uk/project-pages/unit-costs/unit-costs-2015/

12. Kaur G, English C, Hillier S. Physiotherapists systematically overestimate the amount of time stroke survivors spend engaged in active therapy rehabilitation: An observational study. J Physiother. 2013 Mar;59(1):45–51.

13. Ball J, Philippou J, Pike G, Sethi J. Survey of district and community nurses in 2013. Report to the Royal College of Nursing. 2014.

14. The College of Podiatry. To determine the current patient population managed by UK podiatrists and the evidence available to support the effectiveness of podiatric contributions to health and wellbeing. 2015.

15. Pomerantz AS, Corson JA, Detzer MJ. The challenge of integrated care for mental health: Leaving the 50 minute hour and other sacred things. J Clin Psychol Med Settings. 2009 Mar;16(1):40–6.

16. Curtis L, Burns A. Unit Costs of Health and Social Care 2017 [Internet]. Personal Social Services Research Unit, University of Kent, Canterbury; 2017. Available from: https://www.pssru.ac.uk/project-pages/unit-costs/unit-costs-2017/

17. NHS Improvement. Reference costs 2017/18 [Internet]. 2018. Available from: https://improvement.nhs.uk/resources/reference-costs/#rc1718
